# Circulating lipoprotein(a) levels and steatotic liver disease related to metabolic dysfunction in adults: an updated systematic review and meta-analysis

**DOI:** 10.3389/fnut.2026.1778393

**Published:** 2026-05-08

**Authors:** Attia Mustafa, Alexander Dallaway, Lukasz Lagojda, Chris Kite, Farah Abdelhameed, Kamaljit Kaur Chatha, Anirudh Suresh, Harpal S. Randeva, Ioannis Kyrou

**Affiliations:** 1 Warwickshire Institute for the Study of Diabetes, Endocrinology and Metabolism (WISDEM), University Hospitals Coventry and Warwickshire NHS Trust, Coventry, United Kingdom; 2 Warwick Medical School, University of Warwick, Coventry, United Kingdom; 3 Institute for Cardiometabolic Medicine, University Hospitals Coventry and Warwickshire NHS Trust, Coventry, United Kingdom; 4 Department of Internal Medicine, Faculty of Medicine, Omar Al-Mukhtar University, Al-Bayda, Libya; 5 School of Health and Wellbeing, Faculty of Education, Health and Wellbeing, University of Wolverhampton, Wolverhampton, United Kingdom; 6 Sheffield Centre for Health and related Research (SCHARR), School of Medicine and Population Health, University of Sheffield, Sheffield, United Kingdom; 7 Division of Public Health, Sport and Wellbeing, Faculty of Health, Medicine and Society, University of Chester, Chester, United Kingdom; 8 Department of Biochemistry and Immunology, University Hospitals Coventry and Warwickshire NHS Trust, Coventry, United Kingdom; 9 Institute of Precision Diagnostics and Translational Medicine, University Hospitals Coventry and Warwickshire NHS Trust, Coventry, United Kingdom; 10 Discoveries in Life Sciences Research Centre, Coventry University, Coventry, United Kingdom; 11 Aston Medical School, College of Health and Life Sciences, Aston University, Birmingham, United Kingdom; 12 College of Health, Psychology and Social Care, University of Derby, Derby, United Kingdom

**Keywords:** lipoprotein(a), Lp(a), MAFLD, MASLD, metabolic dysfunction-associated fatty liver disease, metabolic dysfunction-associated steatotic liver disease, NAFLD, non-alcoholic fatty liver disease

## Abstract

**Background:**

Metabolic dysfunction-associated steatotic liver disease (MASLD), previously defined as non-alcoholic fatty liver disease (NAFLD) or metabolic dysfunction-associated fatty liver disease (MAFLD), is the leading cause of chronic liver disease worldwide, with a pathophysiological spectrum ranging from steatosis to steatohepatitis and fibrosis. Lipoprotein(a) [Lp(a)] is an atherogenic lipoprotein, which is associated with increased cardiovascular disease (CVD) risk and has been recently reported as a potential biomarker for MASLD. This systematic review and meta-analysis aimed to present an updated evidence synthesis on the potential link between circulating Lp(a) concentrations and this prevalent hepatic disease in adults.

**Methods:**

PubMed, Embase, CINAHL, and Scopus were searched for eligible studies published in English without a date restriction. Risk of bias (RoB) and study quality were assessed using the Revised RoB Assessment Tool for Nonrandomised Studies (RoBANS 2) and the National Institute of Health quality assessment tool, respectively. Three-level meta-regression performed reporting the pooled mean difference of circulating Lp(a) concentrations between adults with MASLD or NAFLD or MAFLD and controls without these conditions.

**Results:**

Twenty-one observational studies were included in this meta-analysis (137,494 cases; 281,261 controls). A three-level meta-analysis resulted in a pooled mean difference of 1.40 mg/dL [95% confidence interval: −2.81, 5.61; *p* = 0.50], indicating no significant difference in circulating Lp(a) concentrations between patients with MASLD or NAFLD or MAFLD and controls. Considerable between-study heterogeneity was observed (*I*^2^ = 95.7%).

**Conclusion:**

These findings provide up-to-date, comprehensive evidence indicating that there are no significant differences in circulating Lp(a) concentrations between adults with metabolic-related steatosis/steatohepatitis and controls. This suggests limited potential for circulating Lp(a) as a diagnostic/prognostic biomarker for MASLD, although this biomarker could still be utilized to assess CVD risk in the context of steatotic liver disease. Future prospective studies are required to further explore the clinical utility of circulating Lp(a) as a biomarker in MASLD, particularly for long-term CVD outcomes.

**Systematic review registration:**

https://www.crd.york.ac.uk/PROSPERO/view/CRD42024607750, Identifier: CRD42024607750.

## 1 Introduction

Non-alcoholic fatty liver disease (NAFLD) is a chronic hepatic condition with a growing global prevalence, impacting an estimated 25–38% of the general population globally (1). In 2020, the term metabolic dysfunction-associated fatty liver disease (MAFLD) was introduced to replace the NAFLD nomenclature (2), whilst, in 2023, this nomenclature was further revised to metabolic dysfunction-associated steatotic liver disease (MASLD) to both emphasize the central role of cardio-metabolic dysfunction in the underlying pathophysiology and abolish the potential stigma linked to NAFLD (3). Although there are differences in the applied diagnostic criteria for each of these terms, NAFLD, MAFLD, and MASLD represent the same hepatic pathology associated with metabolic dysfunction (4), which ranges from simple steatosis to steatohepatitis [metabolic dysfunction-associated steatohepatitis (MASH), previously referred to as non-alcoholic steatohepatitis (NASH)] and fibrosis (4, 5). In addition to the hepatic component, a substantial body of evidence consistently shows that this highly prevalent hepatic disease is further linked to a markedly increased risk of cardiovascular disease (CVD) (6). Notably, evidence indicates that MASLD is closely associated with CVD, with underlying mechanisms involving structural, electrical, and functional alterations of the heart (7). These changes contribute to the development and progression of major cardiovascular events, including coronary artery disease (CAD), atrial fibrillation (AF), and the development of new-onset heart failure (HF) (7).

CVD remains the world’s leading cause of mortality and long-term disability (8). Despite effective management of lowering low-density lipoprotein cholesterol (LDL-C) and other modifiable risk factors, lipoprotein(a) [Lp(a)] is considered an important contributor to residual cardiovascular risk (9). Lp(a), first identified by the Norwegian physician Kare Berg in 1963 (10), is synthesized primarily in the liver and exhibits a unique, complex structure comprising of two main subunits, with an LDL-C-like molecule containing apolipoprotein B linked by a disulfide bond to apolipoprotein (a) (11–14). Compelling research has highlighted that Lp(a) exhibits significant atherogenic, thrombogenic, and pro-inflammatory properties (15), with a significant association between elevated circulating Lp(a) concentrations and the presence of clinical atherosclerosis (16–21). Accordingly, increased circulating Lp(a) concentrations have been linked to increased risk of atherosclerotic cardiovascular disease, independently of traditional risk factors (22, 23). Of note, due to the close link between lipoprotein metabolism and hepatic lipid homeostasis, steatotic liver disease has been shown to significantly alter the circulating lipoprotein profile, contributing to dyslipidaemia which serves as a key driver of CVD risk (24). In this context, several clinical studies have explored the relationship between Lp(a) and steatotic liver disease related to metabolic dysfunction. However, the existing data are conflicting (25–45), with some studies reporting elevated circulating Lp(a) concentrations in patients with steatotic liver disease related to metabolic dysfunction (25, 26, 28–31), while other studies have reported the opposite or a lack of significant differences (27, 32–45). As such, the aim of the present systematic review and meta-analysis was to meta-analyse the existing relevant data and present an updated and comprehensive evidence synthesis of the existing clinical studies comparing circulating Lp(a) concentrations in adults with MASLD or NAFLD or MAFLD and controls without such a hepatic pathology.

## 2 Methods

### 2.1 Protocol and registration

This systematic review was prospectively registered with the International Prospective Register of Systematic Reviews (PROSPERO) (46), with the registration number CRD42024607750. The reporting of the present systematic review and meta-analysis adhered to the Preferred Reporting Items for Systematic Reviews and Meta-Analyses (PRISMA) guidelines (PRISMA checklist included in Supplementary Table 1) (47).

### 2.2 Search strategy and information retrieval

PubMed, Embase, CINAHL, and Scopus were searched from inception to February 2025 for articles published in English. To ensure comprehensive identification of relevant eligible studies, additional searches were carried out in ClinicalTrials.gov and the reference lists of relevant publications. Our search strategy included a combination of controlled vocabulary and keywords for the terms ‘NAFLD/MASLD/MAFLD’ and ‘Lipoprotein a’. The search strategy applied to PubMed is presented in Table 1, with database-specific adaptations detailed in Supplementary Table 2.

**Table 1:** Search strategy of the present systematic review for PubMed.

(“Lipoprotein a” or “lipoprotein(a)” or “lipoprotein (a)” or “apoprotein a” or “apolipoprotein(a)” or “apolipoprotein (a)” or “apolipoprotein a” or “apolipoprotein(a)” or “apolipoprotein (a)” or “Lp a” or “Lp(a)” or “Lp (a)” or “apo a” or “apo(a)” or “apo (a)” or “APOA5”) AND (“metabolic associated fatty liver disease”[Title/Abstract] or MAFLD[Title/Abstract] or “metabolic dysfunction associated fatty liver disease”[Text Word] or “non-alcoholic fatty liver disease”[MeSH Terms] or NAFLD[Title/Abstract] or “non-alcoholic fatty liver disease”[Text Word] or “non-alcoholic steatohepatitis”[Text Word] or NASH[Text Word] or “metabolic dysfunction-associated steatotic liver disease”[Text Word] or “fatty liver”[Title] or MASLD[Text Word])

### 2.3 Eligibility criteria

The eligibility criteria were defined using the Population, Exposure, Comparison, Outcome (PECO) framework (48, 49). All observational study designs which assessed circulating Lp(a) concentrations in adults (≥18 years of age) diagnosed with MASLD or NAFLD or MAFLD in comparison to control groups without such a hepatic pathology were eligible for inclusion. No limitations were applied on the year of publication or the type of healthcare setting. Studies published in languages other than English were excluded. Studies on Lp(a) in pediatric populations, or including other hepatic conditions (e.g., alcoholic hepatitis, chronic hepatitis, autoimmune hepatitis, and hepatocellular carcinoma) were excluded. Editorials, case reports, case series, any type of review articles, interventional studies, expert opinion manuscripts, and commentaries, as well as animal studies, were also excluded.

### 2.4 Study selection and data extraction

Two reviewers (AM and LL) completed the study selection and data extraction independently. The screening of both titles/abstracts and full-text were conducted according to predefined inclusion criteria and were performed using the Rayyan software (50). Relevant data were extracted using a standardized data extraction form, which included data on the country of origin, study design, sample size, sex, age, body mass index (BMI), circulating Lp(a) concentrations, method of Lp(a) measurement, and key outcomes related to the association between Lp(a) and MASLD or NAFLD or MAFLD. Where necessary, the author(s) of included studies were contacted to provide and/or confirm study data which were relevant to the present meta-analysis.

### 2.5 Risk of bias and quality assessment

The risk of bias (RoB) and quality assessment were completed independently by two reviewers (AM and LL). The RoB was assessed using the Revised RoB Assessment Tool for Nonrandomised Studies (RoBANS 2), which evaluates eight key domains (i.e., comparability of the target group, target group selection, confounders, measurement of exposure, blinding of assessors, outcome assessment, incomplete outcome data, and selective outcome reporting) (51). The quality assessment was conducted using the National Institute of Health (NIH) Study Quality Assessment Tool (52).

### 2.6 Statistical analysis

A meta-analysis of eligible studies was conducted using a random-effects model to account for potential heterogeneity among study results. Where necessary, unit conversions were performed to standardize outcome measures across studies for meta-analysis, such as converting mg/L to mg/dL or g/L to mg/dL. For Lp(a) concentrations reported in molar units (nmol/L), values were converted to mass units (mg/dL) (28, 37, 42–44) by applying a standard conversion factor of 0.465 (53). Data presented as median and interquartile range (IQR) were used to estimate mean and standard deviation (SD) values using the methods outlined by Luo et al. (54) and Wan et al. (55).

A random effects three-level meta-analytical model was fitted to the data of eligible studies, using restricted maximum likelihood (REML) as the variance estimator. Studies that contributed more than one effect size or used the same participants/cohort were clustered at the third level (i.e., between-study variance). The second level modeled variance between individual effect sizes within studies (i.e., within-study variance), and the first level accounted for sampling variance (56). The three-level meta-analysis was conducted using the “metaviz” and metafor packages (57, 58) within the RStudio environment (version 2024.04.2 + 764; R version 4.4.1). Adopting a three-level approach enabled a detailed examination of outcome differences and managed statistical dependency arising from studies contributing multiple effect sizes. Given the complexity of the extracted data, traditional meta-analytical approaches were deemed unsuitable, as they underestimate variance within and overestimate variance between clusters (59). Meta-regression and subgroup analyses were performed to investigate the effect of potential moderators separately, with Knapp-Hartung adjustments to calculate the 95% Confidence Interval (CI) around the pooled effect (60). Categorical moderators included the diagnostic method of MASLD, NAFLD and MAFLD, the measurement method of Lp(a), disease type/diagnosis (i.e., MASLD/MASH, NAFLD/NASH, MAFLD/MASH). The BMI and the difference in the percentage of females that made up the patient versus the control group were also included as potential continuous moderators.

Heterogeneity was investigated at the within-cluster (level 2) and between-cluster (level 3) levels. The *I*^2^ statistic was used to express the percentage of total variation in effect estimates attributable to true heterogeneity rather than sampling error, with interpretation following the guidelines by Deeks et al.: 0–40% may indicate minimal heterogeneity, 30–60% moderate heterogeneity, 50–90% substantial heterogeneity, and 75–100% considerable heterogeneity (61).

Sensitivity analyses were conducted to also explore sources of heterogeneity and identify influential studies. Sensitivity analyses included the removal of studies assessed as “poor” and “fair” quality based on the NIH quality criteria (52). Additionally, outliers were investigated using a leave-one-out analysis and visual inspection of Baujat plots. However, due to statistical dependency and the structure of the data, outliers were considered at the cluster level. Therefore, to explore the contribution of study clusters to between-study heterogeneity, to identify outliers and to evaluate the robustness of the summary effect estimate, cluster-level diagnostics were performed. A clustered Baujat plot was constructed to assess the relative contribution of each cluster to overall heterogeneity and to the pooled mean difference. Rather than individual study effects, this approach summarized effects at the cluster level. Clusters (or individual studies that formed their own cluster of k = 1) located away from the others were considered influential, contributing disproportionately to both heterogeneity and the pooled effect estimate.

To confirm outliers, a leave-one-cluster-out analysis was conducted. Each cluster (or individual studies that formed their own cluster of k = 1) was sequentially excluded from the dataset, and the three-level random-effects meta-analysis was refitted. For each refitted model, the revised pooled mean difference and corresponding 95% CI were extracted. Changes in heterogeneity were evaluated using the estimated variance components at the within-cluster (Level 2) and between-cluster (Level 3) levels, expressed as proportions of total variance (*I*^2^).

Publication bias was evaluated using an Egger’s-type regression applied to the three-level model, where effect sizes were regressed on their standard errors to assess small-study effects (58, 62). A power-enhanced sunset funnel plot was also generated using the “metaviz” package (63) to visually represent the statistical power of the included studies relative to the pooled effect size (64). To evaluate the evidential value of the included studies, a p-curve analysis was conducted using the dmetar package (65). This method assesses whether the pattern of statistically significant results aligns with the presence of a genuine underlying effect, rather than reflecting potential publication bias or data manipulation (66, 67).

## 3 Results

### 3.1 Study selection

**Figure 1:**
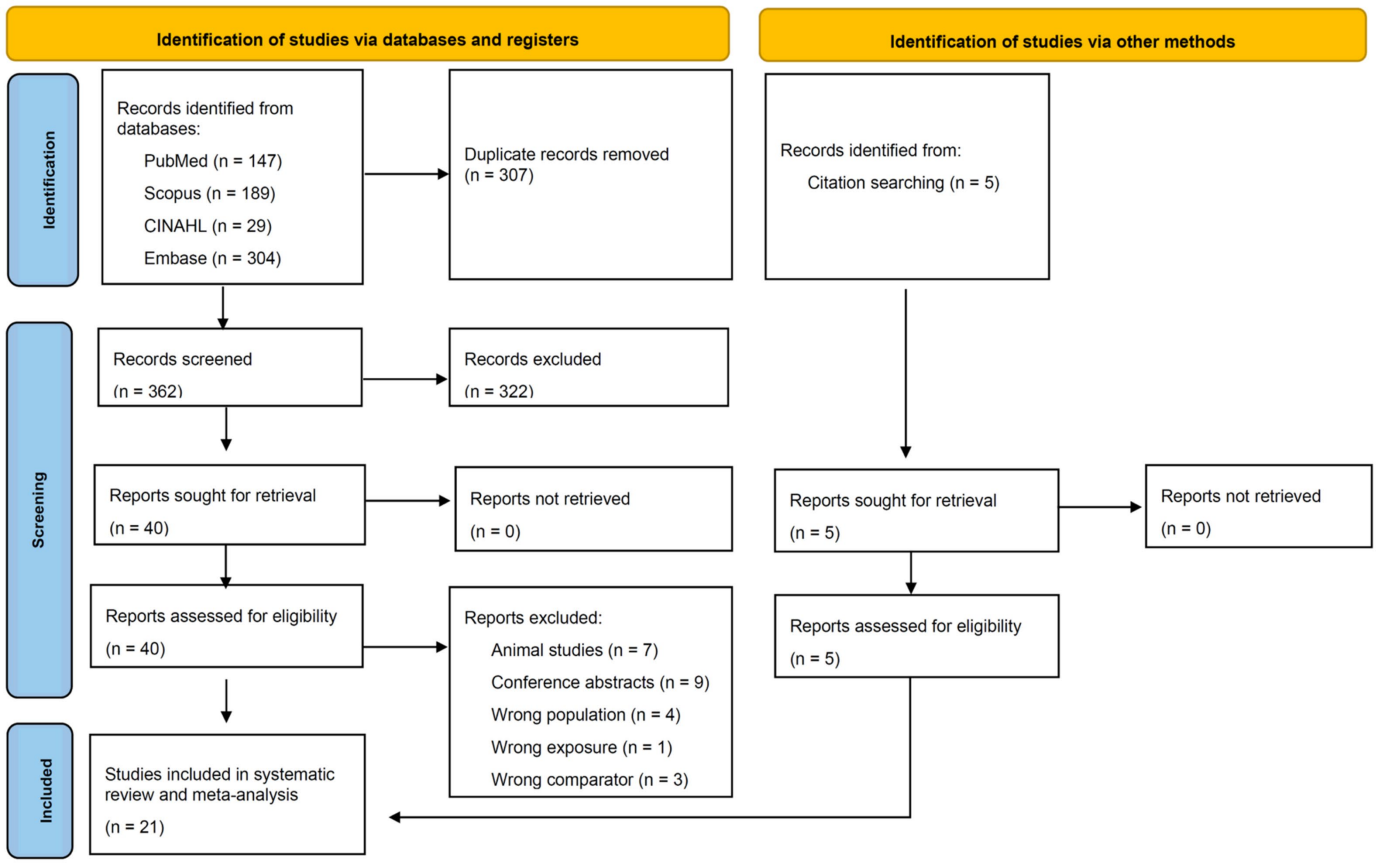
PRISMA flow diagram for the identification of eligible studies included in the present systematic review and meta-analysis.

The database search results and study selection process are presented in Figure 1. A total of 669 studies were identified. After removing 307 duplicate records, 362 studies remained for title and abstract screening. Of these, 322 studies were excluded for not meeting the inclusion criteria, leaving 40 studies eligible for full-text review. During the full-text review, 24 studies were excluded with documented reasons, as listed in Figure 1, resulting in 16 studies eligible for inclusion. Additionally, five eligible studies were identified through citation searching and reference list screening. This resulted in a total of 21 eligible studies for this systematic review (25–45).

All 21 included studies were observational and had a control group of patients without metabolic-related steatosis/steatohepatitis (Table 2). Most studies were published from South Korea (*n* = 6) (34, 35, 37, 40, 44, 45) and China (*n* = 6) (26, 27, 29, 31, 38, 39), while there were also two studies from Turkey [both by Koruk et al. (25, 32)], and one each from India (33), Malaysia (28), Serbia (36), Belgium (43), Colombia (30) and Egypt (41). Of note, the study by Ye et al. (27) reported data from more than one cohorts/studies from different countries (i.e., two Chinese cohorts and two groups from the UK Biobank), while the study by Fan et al. (42) reported data from both a multi-centre study and the UK Biobank cohort (Table 2). Supplementary Figure 1 displays a global map with the countries of origin for the cohorts included in the 21 eligible studies of this systematic review. The overall number of participants in these studies (*n* = 418,755 adults) included 137,494 cases with MASLD or NAFLD/NASH or MAFLD and 281,261 controls. Of the included studies, 12 applied the NAFLD diagnosis (*n* = 12,977) (26, 28, 29, 31, 34–37, 41, 43–45), whilst four studies (25, 26, 32, 33) further defined the diagnosis as NASH (*n* = 95). The MAFLD diagnosis was applied in four studies (*n* = 119,312) (27, 38–40), while the most recent term/nomenclature of MASLD was applied in two eligible studies (*n* = 5,110) (30, 42).

**Table 2:** Key characteristics and pertinent findings of the 21 eligible studies included in the present systematic review.

Author (Year) (ref.)	Study characteristics	Participant characteristics NAFLD/NASH; MAFLD; MASLD	Participant characteristics controls	Key findings
Koruk et al. (2003) (25)	Country: Turkey Design: Cross-Sectional Study Lp(a) measurement method: Nephelometry Diagnosis: NASH Diagnostic Method: Liver Biopsy	*N*: 36 Sex: 75.0% Male Age: 44 ± 7.1 years BMI: 28.5 ± 3.3 kg/m^2^	*N*: 32 Sex: 68.75% Male Age: 40 ± 10.3 years BMI: 27.2 ± 4.1 kg/m^2^	No significant difference in serum Lp(a) levels between the NASH (13.09 ± 9.61 mg/dL) and control (12.01 ± 7.50 mg/dL) groups (*p* = 0.61).
Koruk et al. (2003) (32)	Country: Turkey Design: Cross-Sectional Study Lp(a) measurement method: Nephelometry Diagnosis: NASH Diagnostic Method: Liver Biopsy	N: 18 Sex: 72.22% Male Age: 44 ± 7.1 years BMI: 28.5 ± 3.3 kg/m^2^	N: 16 Sex: 68.75% Male Age: 40 ± 10.3 years BMI: 27.2 ± 4.1 kg/m^2^	No significant difference in serum Lp(a) levels between the NASH (13.06 ± 9.61 mg/dL) and control (12.01 ± 7.5 mg/dL) group (*p* = 0.61) [The study by Koruk et al. (32) appears to report similar data to the study by Koruk et al. (25). Attempts to contact the study authors by email to clarify this point were not successful].
Zhang et al. (2020) (26)	Country: China Design: Cross-Sectional Study Lp(a) measurement method: latex-enhanced immunoturbidimetry Diagnosis: NAFLD Diagnostic method: Ultrasonography Diagnosis: NASH Diagnostic method: Liver Biopsy	NAFLD – *N*: 63 Sex: 66.67% Male Age: 38.92 ± 9.80 years BMI: No data reported NASH – *N*: 23 Sex: 69.57% Male Age: 40.63 ± 10.65 years BMI: No data reported	*N*: 81 Sex: 65.43% Male Age: 38.94 ± 5.40 years BMI: No data reported *N*: 81 Sex: 65.43% Male Age: 38.94 ± 5.40 years BMI: No data reported	No significant difference in serum Lp(a) levels between the NAFLD (15.36 ± 19.93 mg/dL) and control (12.43 ± 14.95 mg/dL) groups (*p* = 0.38) Serum Lp(a) levels were significantly higher in patients with NASH (33.01 ± 44.09 mg/dL) compared to those with NAFLD (15.36 ± 19.93 mg/dL) (*p* = 0.04) [The study authors were contacted and provided the Lp(a) values]
Ye et al. (2022) (27)	Country: China Design: Two phases with different cohorts were included in this study; Phase 1 included a cross-sectional study of Han Chinese patients with MAFLD (diagnosis based on MRI-PDFF) for cluster analyses; Phase 2 validated the prognostic value of the clusters identified in the Phase 1 study using the retrospective longitudinal data of a Chinese cohort of patients with MAFLD (diagnosis based on ultrasonography) and an international UK Biobank cohort of patients with MAFLD [diagnosis based on FLI cut-off values ≥60; whilst a subgroup had MRI-PDFF] Lp(a) measurement method: Immunoturbidimetry Diagnosis: MAFLD (UK Biobank cohort) Diagnostic method: MRI-PDFF Diagnosis: MAFLD (UK Biobank cohort) Diagnostic method: FLI Diagnosis: MAFLD (Chinese cohort; Phase 2 validation cohort) Diagnostic method: Ultrasonography Diagnosis: MAFLD (Chinese cohort; Phase 1 cohort) Diagnostic method: MRI-PDFF	MAFLD (UK Biobank cohort with MRI-PDFF data) *N*: 1,010 Sex: 58.8% Male Age: 55.9 ± 7.4 years BMI: 29.3 ± 4.3 kg/m^2^ MAFLD (UK Biobank cohort with FLI data) *N*: 106,408 Sex: 63.8% Male Age: 56.7 ± 7.9 years BMI: 31.3 ± 4.4 kg/m^2^ MAFLD (Chinese cohort with ultrasonography data) *N*: 3,494 Sex: 61.0% Male Age: 46.1 ± 13.6 years BMI: 25.9 ± 3.7 kg/m^2^ MAFLD (Chinese cohort with MRI-PDFF data) *N*: 1,038 Sex: 74.5% Male Age: 41.3 ± 12.4 years BMI: 26.8 ± 3.6 kg/m^2^	*N*: 3,601 Sex: 44.0% Male Age: 55.4 ± 7.7 years BMI: 25.9 ± 3.9 kg/m^2^ *N*: 197,733 Sex: 35.2% Male Age: 56.1 ± 8.2 years BMI: 24.9 ± 2.8 kg/m^2^ *N*: 6,957 Sex: 60.4% Male Age: 46.7 ± 12.5 years BMI: 22.6 ± 3.4 kg/m^2^ *N*: 143 Sex: 58.3% Male Age: 41.6 ± 11.2 years BMI: 24.8 ± 2.8 kg/m^2^	Through cluster analysis of demographic and metabolic data (age, HbA1c, TC, triglyceride, HDL-C and Lp(a) levels) of the cross-sectional Phase 1 cohort, this study identified five clusters of homogenous phenotypes among the patients with MAFLD: (1) Cluster 1 (~40% of the clustered participants) was named as mild obesity and dyslipidemia related MAFLD, and was characterized by slightly higher BMI, serum triglycerides, TC/HDL-C ratio, and Lp(a) levels than those of the controls; (2) Cluster 2 (28.2%) was termed as age-related MAFLD and was characterized by the greatest mean age, relatively higher triglyceride levels and higher TC/HDL-C ratio, and HOMA-IR than Cluster 1; (3) Cluster 3 (16.7%) was named severe insulin resistance related MAFLD and was characterized by severe obesity and an extremely high HOMA-IR; (4) Cluster 4 (9.4%) was named as high Lp(a)-related MAFLD and was characterized by extremely high Lp(a) levels, but relatively lower triglyceride levels, TC/HDL-C ratio and HOMA-IR than the other clusters; (5) Cluster 5 (5.7%) was named as severe mixed hyperlipidemia-related MAFLD and was characterized by an extremely high TC/HDL-C ratio, and triglyceride levels, as well as relatively high Lp(a) levels, BMI, and HOMA-IR. The study analyses showed that classification into these clusters is superior regarding prognosis and the related complication risks particularly for the long-term T2DM and CHD risks. These findings suggest that MAFLD elevated serum Lp(a) levels are associated with a higher CHD risk, although there may be milder hepatic steatosis, inferring that Lp(a) is more useful to predict the development of CVD in MAFLD
Wu et al. (2021) (31)	Country: China Design: Retrospective Cohort Study Lp(a) measurement method: Immunoturbidimetry Diagnosis: NAFLD Diagnostic method: Ultrasonography	*N*: 3,519 Sex: 61.0% Male Age: 46.1 ± 14.5 years BMI: 25.9 ± 4.0 kg/m^2^	*N*: 6,934 Sex: 60.4% Male Age: 46.6 ± 14.4 years BMI: 22.6 ± 3.0 kg/m^2^	Serum Lp(a) levels were significantly higher in the NAFLD (26.7 ± 28.8 mg/dL) compared to the control (22.7 ± 20.6 mg/dL) group (*p* = 0.027)
Wu et al. (2021) (38)	Country: China Design: Cross-Sectional Study Lp(a) measurement method: Immunoturbidimetry Diagnosis: MAFLD Diagnostic method: Ultrasonography/MRI-PDFF	*N*: 1,038 Sex: 74.6% Male Age: 41.4 ± 12.2 years BMI: 26.8 ± 3.6 kg/m^2^	*N*: 144 Sex: 58.3% Male Age: 41.6 ± 11.2 years BMI: 24.8 ± 2.8 kg/m^2^	No significantly different serum Lp(a) levels between the MAFLD (10.02 ± 10.02 mg/dL) and the control (12.20 ± 12.58 mg/dL) group (*p* = 0.46)
Wu et al. (2022) (39)	Country: China Design: Cross-Sectional Study Lp(a) measurement method: Immunoturbidimetry Diagnosis: MAFLD Diagnostic method: Ultrasonography/MRI	*N*: 1,346 Sex: 64.9% Male Age: 47.7 ± 13.8 years BMI: 26.1 ± 3.0 kg/m^2^	*N*: 3,002 Sex: 56.9% Male Age: 45.7 ± 12.7 years BMI: 23.2 ± 2.9 kg/m^2^	No significantly different serum Lp(a) levels are presented in Table 1 of this publication between the MAFLD (14.39 ± 15.21 mg/dL) and the control (13.84 ± 12.75 mg/dL) group (*p* = 0.11). However, the abstract of this publication reports that participants without MAFLD had higher Lp(a) levels than patients with MAFLD with or without advanced fibrosis (both *p*-values <0.05) [Note: Attempts to contact the study authors by email to clarify this point were not successful]
Nam et al. (2016) (35)	Country: South Korea Design: Cross-Sectional Study Lp(a) measurement method: Nephelometry Diagnosis: NAFLD Diagnostic method: Ultrasonography	*N*: 975 Sex: 78.0% Male Age: 52.81 ± 9.18 years BMI: 26.35 ± 2.97 kg/m^2^	*N*: 1,267 Sex: 54.0% Male Age: 52.88 ± 10.12 years BMI: 22.88 ± 2.94 kg/m^2^	Serum Lp(a) levels were significantly lower in the NAFLD (12.94 ± 11.95 mg/dL) compared to the control (15.6 ± 13.35 mg/dL) group (*p* < 0.01) [Note: Data from patients with mild, moderate, and severe NAFLD were aggregated]
Nam et al. (2024) (45)	Country: South Korea Design: Retrospective Longitudinal Study Lp(a) measurement method: Nephelometry Diagnosis: NAFLD Diagnostic method: Ultrasonography	*N*: 352 Sex: 96.59% Male Age: 52.2 ± 9.0 years BMI: 24.3 ± 2.6 kg/m^2^	*N*: 1,149 Sex: 94.69% Male Age: 53.4 ± 9.8 years BMI: 23.3 ± 2.5 kg/m^2^	Serum Lp(a) levels were significantly lower in the NAFLD (0.156 ± 0.131 mg/dL) compared to the control (0.215 ± 0.234 mg/dL) group (*p* < 0.001)
Choe et al. (2013) (34)	Country: South Korea Design: Cross-Sectional Study Lp(a) measurement method: Nephelometry Diagnosis: NAFLD Diagnostic method: Ultrasonography	*N*: 2,554 Sex: 82.6% Male Age: 42.49 ± 5.13 years BMI: 25.63 ± 2.61 kg/m^2^	*N*: 6,608 Sex: 44.6% Male Age: 41.64 ± 4.88 years BMI: 22.38 ± 2.53 kg/m^2^	Serum Lp(a) levels were significantly lower in the NAFLD (23.80 ± 22.94 mg/dL) compared to the control (27.30 ± 25.42 mg/dL) group (*p* < 0.001)
Jung et al. (2020) (37)	Country: South Korea Design: Cross-Sectional Study Lp(a) measurement method: High sensitivity immunoturbidimetry Diagnosis: NAFLD Diagnostic method: Ultrasonography	*N*: 3,030 Sex: 77.3% Male Age: 37.93 ± 6.15 years BMI: 24.04 ± 2.41 kg/m^2^	*N*: 19,504 Sex: 48.9% Male Age: 37.67 ± 5.85 years BMI: 21.94 ± 2.44 kg/m^2^	Serum Lp(a) levels were significantly lower in the NAFLD (32.69 ± 28.6 mg/dL) compared to the control (34.37 ± 29.96 mg/dL) group (*p* = 0.001)
Fan et al. (2024) (42)	Country: United Kingdom Design: Of the reported studies by Fan et al., Study 2, which is a prospective cohort study (UK Biobank cohort study), was eligible for inclusion Lp(a) measurement method: Immunoturbidimetry Diagnosis: MASLD Diagnostic method: MRI-PDFF	*N*: 5,037 Sex: 40.0% Male Age: 55.42 ± 7.20 years BMI: 29.30 ± 4.17 kg/m^2^	*N*: 17,197 Sex: 56.2% Male Age: 55.21 ± 7.52 years BMI: 25.63 ± 3.72 kg/m^2^	Serum Lp(a) levels were significantly lower in the MASLD (8.23 ± 23.76 mg/dL) compared to the control (8.92 ± 22.32 mg/dL) group (*p* < 0.001)
Sukahri et al. (2021) (28)	Country: Malaysia Design: Cross-Sectional Study Lp(a) measurement method: Nephelometry Diagnosis: NAFLD Diagnostic method: Ultrasonography	*N*: 90 Sex: 67.8% male Age: 51.3 ± 13.0 years BMI: 36.2 ± 9.4 kg/m^2^	*N*: 20 Sex: 55.0% male Age: 45.0 ± 17.0 years BMI: 27.7 ± 5.6 kg/m^2^	Serum Lp(a) levels were significantly higher in the NAFLD (39.7 ± 24.44 mg/dL) compared to the control (10.9 ± 11 mg/dL) group (*p* < 0.001)
Lin et al. (2021) (29)	Country: China Design: Cross-Sectional Study Lp(a) measurement method: Not reported Diagnosis: NAFLD Diagnostic method: Ultrasonography	*N*: 2015 Sex: 54.5% Male Age: 64.4 ± 18.9 years BMI: 24.3 ± 5.5 kg/m^2^	*N*: 6916 Sex: 61.4% Male Age: 66.1 ± 19.2 years BMI: 21.6 ± 3.6 kg/m^2^	Serum Lp(a) levels were significantly higher in the NAFLD (35.33 ± 4.25 mg/dL) compared to the control (28.93 ± 3.23 mg/dL) group (*p* = 0.007)
Rossi et al. (2024) (30)	Country: Colombia Design: Cross-Sectional Study Lp(a) measurement method: Not reported Diagnosis: MASLD Diagnostic method: Ultrasonography	*N*: 73 Sex: 38.9% Male Age: 48.7 ± 11.3 years BMI: 29.6 ± 4.33 kg/m^2^	*N*: 53 Sex: 54.9% Male Age: 50.6 ± 14.0 years BMI: 25.6 ± 3.78 kg/m^2^	The FIB-4 score had a weak but statistically significant inverse correlation with the Lp(a) levels (*r* = −0.241, *p* = 0.048) [Note: The age, BMI and Lp(a) values were provided by the study authors]
Priya et al. (2010) (33)	Country: India Design: Cross-Sectional Study Lp(a) measurement method: Not reported Diagnosis: NASH Diagnostic method: Liver Biopsy	*N*: 18 Sex: 55.56% Male Age: No data BMI: 29.5 ± 3.5 kg/m^2^	*N*: 28 Sex: 64.29% Male Age: No data BMI: 27.2 ± 4.1 kg/m^2^	No significantly different serum Lp(a) levels between the NASH (13.56 ± 7.66 mg/dL) and control (12.01 ± 7.50 mg/dL) group (*p* > 0.05)
Kojić et al. (2018) (36)	Country: Serbia Design: Cross-Sectional Study Lp(a) measurement method: Immunoturbidimetry Diagnosis: NAFLD Diagnostic method: Ultrasonography	*N*: 44 Sex: 52.27% Male Age: 42.34 ± 6.57 years BMI: 34.38 ± 9.73 kg/m^2^	*N*: 33 Sex: 36.36% Male Age: 39.36 ± 6.40 years BMI: 28.05 ± 4.79 kg/m^2^	No significantly different serum Lp(a) levels between the NAFLD (25.0 ± 30.0 mg/dL) and the control (18.0 ± 28.0 mg/dL) group (*p* = 0.095)
Park et al. (2023) (40)	Country: South Korea Design: Cross-Sectional Study Lp(a) measurement method: Not reported Diagnosis: MAFLD Diagnostic method: Ultrasonography	*N*: 4,978 Sex: 84.7% Male Age: 46.0 ± 0.0 years BMI: 26.5 ± 0.14 kg/m^2^	*N*: 9,441 Sex: 57.70% Male Age: 45.35 ± 0.74 years BMI: 22.73 ± 0.07 kg/m^2^	Serum Lp(a) levels were significantly lower in the MAFLD (7.7 ± 0.48 mg/dL) compared to the control (9.94 ± 0.48 mg/dL) group (*p* < 0.0001)
El-Gazar et al. (2022) (41)	Country: Egypt Design: Cross-Sectional Study Lp(a) measurement method: Nephelometry Diagnosis: NAFLD Diagnostic method: not clearly described in the methods of this paper	*N*: 50 Sex: 76.0% Male Age: 54.32 ± 16.79 years BMI: No data	*N*: 50 Sex: 82% Male Age: 55.10 ± 11.65 years BMI: No data	Serum Lp(a) levels were significantly lower in the NAFLD (11.69 ± 2.36 mg/dL) compared to the control (14.60 ± 1.97 mg/dL) group (*p* < 0.001)
Hermans et al. (2022) (43)	Country: Belgium Design: Cross-Sectional Study Lp(a) measurement method: Not reported Diagnosis: NAFLD Diagnostic method: Ultrasonography	*N*: 235 Sex: 64.0% Male Age: 69.0 ± 13.0 years BMI: 29.7 ± 5.7 kg/m^2^	*N*: 171 Sex: 58.0% Male Age: 70.0 ± 15 years BMI: 25.9 ± 4.9 kg/m^2^	No statistically significant differences were observed in serum Lp(a) levels between participants with NAFLD without atherogenic dyslipidemia (18.33 ± 25.67 mg/dL) and the control group (non-NAFLD participants without atherogenic dyslipidemia) (24.38 ± 32.32 mg/dL), with the p-value reported as not significant.
Lee et al. (2006) (44)	Country: South Korea Design: Cross-Sectional Study Lp(a) measurement method: Immunoturbidimetry Diagnosis: NAFLD Diagnostic method: Ultrasonography	*N*: 50 Sex: 33.3% Male Age: 49.1 ± 12.9 years BMI: 28.2 ± 3.1 kg/m^2^	*N*: 100 Sex: 33.3% Male Age: 47.8 ± 10.5 years BMI: 22.8 ± 2.6 kg/m^2^	Serum Lp(a) levels were significantly lower in the NAFLD (7.94 ± 5.39 mg/dL) compared to the control (12.55 ± 10.65 mg/dL) group (*p* < 0.001) [Note: Lp(a) levels in this publication were reported in nmol/L, as provided by the study authors]

APASL, Asian Pacific Association for the Study of the Liver; BMI, body mass index; CAD, coronary artery disease; CHD, coronary heart disease; CIMT, carotid intima media thickness; CVD, cardiovascular disease; FIB-4 score, Fibrosis-4 Score; HbA1c, Hemoglobin A1c; HDL-C, high-density lipoprotein cholesterol; HOMA-IR, Homeostatic Model Assessment of Insulin Resistance; kg/m^2^, kilograms per square meter; Lp(a), Lipoprotein(a); MAFLD, metabolic dysfunction-associated fatty liver disease; MASLD, metabolic dysfunction-associated steatotic liver disease; mg/dl, milligrams per deciliter; MRI-PDFF, magnetic resonance imaging proton density fat-fraction measurement; N, number; NAFLD, non-alcoholic fatty liver disease; NASH, non-alcoholic steatohepatitis; PAD, peripheral artery disease; T2DM, Type 2 diabetes mellitus; TC, total cholesterol; TC/HDL-C ratio, total cholesterol to HDL cholesterol ratio; USA, United States of America. Data are presented as mean ± standard deviation or percentages.

Across the included studies, the proportion of male participants in the patient (NAFLD/NASH or MAFLD or MASLD) groups ranged from 33.0% (44) to 96.59% (45), whereas in the control groups this ranged from 33.0% (44) to 94.69% (45), indicating a broad distribution of sex across cases and controls. The mean age of individuals in the disease groups varied between 37.93 (37) and 69.0 (43) years, whilst the reported mean BMI ranged from 24.04 (37) to 36.2 (28) kg/m^2^. Respectively, in the control groups the reported mean age ranged from 37.67 (37) to 70.0 (43) years and the BMI from 21.6 (29) to 28.05 (36) kg/m^2^ (Table 2).

The methods used to measure circulating Lp(a) varied among the included studies; namely, nine studies used the immunoturbidimetry method (26, 27, 31, 36–39, 42, 44), and seven studies used the nephelometry method (25, 28, 32, 34, 35, 41, 45), while there were five studies which failed to specify the Lp(a) measurement method (29, 30, 33, 40, 43). Circulating Lp(a) concentrations were reported in mass units in all studies, except for five which reported values in molar units (28, 37, 42–44).

The method for diagnosing steatosis/steatohepatitis also varied across the included studies. Most studies used ultrasonography (26–31, 34–37, 39, 40, 43–45), while a few studies included MRI-assessed cohorts/groups (27, 38, 39), including two cohorts reported by Ye et al. (27) (Table 2). Liver biopsy was performed in four studies (25, 26, 32, 33), while the fatty liver index (FLI) with a cut-off value of ≥60 was used for one of the cohorts (i.e., the UK Biobank cohort) included in the study by Ye et al. (27). The diagnostic method was insufficiently described for the patients with NAFLD in the study by El-Gazar et al. (41). Further details regarding the study design and the pertinent characteristics of each eligible study are summarized in Table 2.

### 3.2 Assessment of risk of bias in included studies

**Figure 2:**
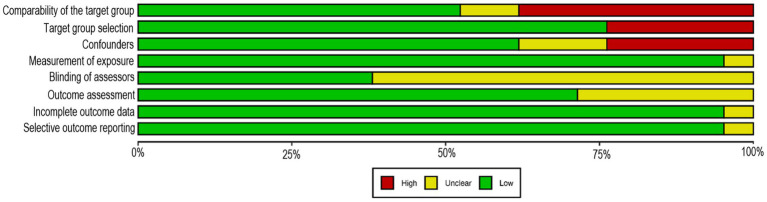
Risk of bias and quality assessment summary. Review of judgments on each domain from the Risk of Bias Assessment Tool for Nonrandomized Studies (RoBANS 2), presented as high (red color), unclear (yellow color), or low (green color) risk across all included studies.

The RoB summary across the eight domains of RoBANS 2 is presented in Figure 2, whilst Supplementary Figure 2 details the RoB assessment for each of the 21 included studies. For the comparability of the target group domain, 11 studies had a low RoB, while two studies had unclear risk, and eight studies had a high risk primarily attributed to significant differences between the patient and control groups. Similarly, in the target group selection domain, most of the included studies (*n* = 16) had a low RoB, while five had a high risk. In the confounding variables domain, 13 of the included studies had a low RoB, while five had a high RoB, and three studies had an unclear risk. While liver biopsy is the gold standard for diagnosing steatosis/steatohepatitis, all included studies, except for one, reported using validated diagnostic methods which are routinely used in clinical practice. Consequently, the risk of performance bias related to exposure assessment was considered low across all studies, except for one study in which the diagnostic method was insufficiently described, resulting in an unclear risk (41). Regarding the blinding of the assessor domain, eight studies had a low RoB, and 13 of the included studies had an unclear RoB. In terms of outcomes assessment, 15 of the included studies had a low RoB, while six had unclear risk. Finally, when assessing the incomplete outcome data and selective outcome reporting, the included studies were judged to have a low RoB for both of these domains, except for the study by Zhang et al. (26), where the RoB was considered unclear due to insufficient information on data handling and outcome reporting.

Supplementary Figure 2 also presents the quality assessment of the included studies based on the NIH Quality Assessment Tool. The majority of included studies (*n* = 15; 71%) were rated as high quality (26–29, 31, 35–40, 42–45), while two studies (*n* = 2; 10%) were rated as fair (33, 34), and four studies (*n* = 4; 19%) were rated as low quality (25, 30, 32, 41). The most common factors contributing to a lower quality rating were the absence of sample size justification, inconsistent management of exposure severity, and inadequate control of confounding variables.

### 3.3 Circulating Lp(a) and NAFLD/NASH or MAFLD or MASLD

Based on the reported study details, it is likely that certain studies had overlapping participants/populations, and, due to this possibility, the corresponding studies were clustered together in the meta-analysis regarding the circulating Lp(a) concentrations in cases and controls. As such, studies were clustered into (i) the Sun Yat-sen University (SYSU), Guangdong, China multicentre cohort/cluster [cohort from Wu et al. 2021 (31), cohort from Lin et al. 2021 (29), and the Phase-2 Chinese cohort (validation cohort) from Ye et al. 2022 (27)]; (ii) the SYSU single-centre cohort/cluster [cohort from Wu et al. 2021 (38), the Phase-1 Chinese cohort from Ye et al. 2022 (27), and Wu et al. 2022 (39)]; (iii) the United Kingdom (UK) Biobank MAFLD cohort/cluster [the two UK Biobank cohorts/groups from Ye et al. 2022 (27), one from Fan et al. 2024 (42)]; (iv) the Gangnam Severance Hospital health-check cohort/cluster [cohorts from Nam et al. 2016 (35), and Nam et al. 2024 (45)]; (v) the Kangbuk Samsung Hospital health-check cohort/cluster [cohorts from Choe et al. 2013 (34) and from Jung et al. 2020 (37)]; (vi) the Atatürk University NASH cohort/cluster [cohorts from the two studies by Koruk et al. 2003a (32) and Koruk et al. 2003b (25)]; and (vii) the West China Hospital cohort/cluster including the groups from the Zhang et al. 2020 (26) study, which represented a single cohort that was categorized to control, NAFLD, and NASH.

**Figure 3:**
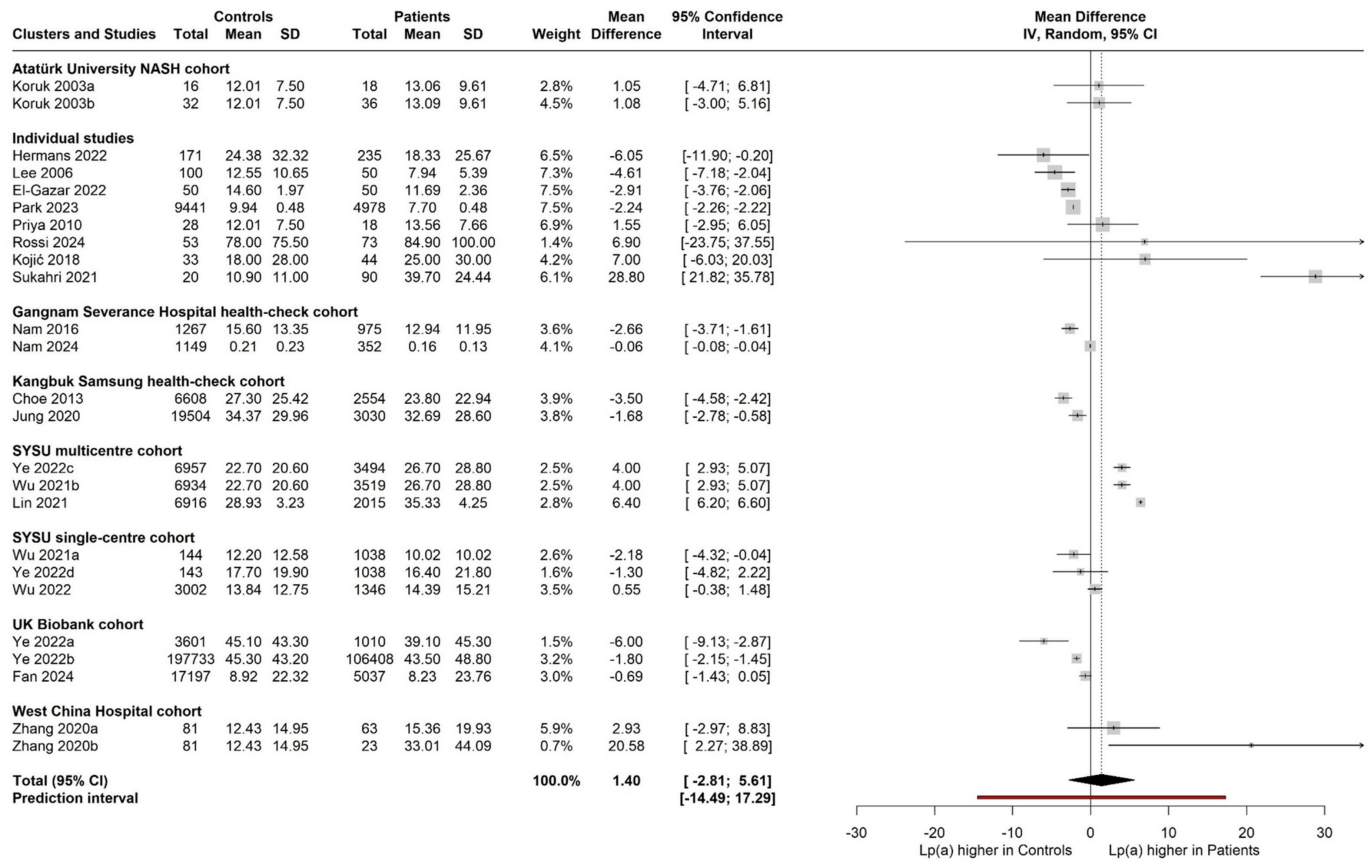
Forest plot with the differences of circulating lipoprotein(a) [Lp(a)] concentrations between cases (adults diagnosed with MASLD or NAFLD or MAFLD or NASH) and controls through a three-level meta-analysis [random-effects model, mean difference]. The cluster column indicates the identifier for the cluster to which each study belongs in the three-level model: (i) the Sun Yat-sen University (SYSU), Guangdong, China multicenter cohort/cluster [Wu 2021b = cohort from Wu et al. 2021 (31), cohort from Lin et al. 2021 (29), and Ye 2022c = the Phase-2 Chinese cohort (validation cohort) from Ye et al. 2022 (27)]; (ii) the SYSU single-centre cohort/cluster [Wu 2021a = cohort from Wu et al. 2021 (38), Ye 2022d = the Phase-1 Chinese cohort from Ye et al. 2022 (27), and cohort from Wu et al. 2022 (39)]; (iii) the United Kingdom (UK) Biobank MAFLD cohort/cluster [Ye 2022a and Ye 2022b = the two UK Biobank cohorts/groups from Ye et al. 2022 (27), and cohort from Fan et al. 2024 (42)]; (iv) the Gangnam Severance Hospital health-check cohort/cluster [cohorts from Nam et al. 2016 (35), and Nam et al. 2024 (45)]; (v) the Kangbuk Samsung Hospital health-check cohort/cluster [cohorts from Choe et al. 2013 (34), and from Jung et al. 2020 (37)]; (vi) the Atatürk University NASH cohort/cluster [cohorts from the two studies by Koruk et al. 2003a (32) and Koruk et al. 2003b (25)]; and (vii) the West China Hospital cohort/cluster including the NAFLD [Zhang 2020a] and NASH [Zhang 2020b] groups from the Zhang et al. 2020 (26) study which represented a single cohort that was categorized to control, NAFLD, and NASH groups.

A random-effects meta-analysis of the 21 eligible studies yielded a pooled mean difference (MD) of 1.31 mg/dL (95% CI: −2.93 to 5.56, *p* = 0.52), indicating no significant difference in Lp(a) levels between patients with NAFLD/NASH or MAFLD or MASLD and those without. Considerable heterogeneity was observed across studies (*I*^2^ = 94.5%, *p* < 0.0001). To address statistical dependency between effect sizes arising from potentially overlapping cohorts, a three-level random-effects model was employed with the aforementioned clusters, resulting in a summary effect of 1.40 mg/dL (MD = 1.40 mg/dL, 95% CI: −2.81 to 5.61, *p* = 0.50), which similarly did not reach statistical significance (Figure 3). The broad prediction interval presented in Figure 3 reflects considerable real-world heterogeneity, and, consequently, the pooled estimate should not be interpreted as evidence of a consistent effect across populations, which significantly limits the clinical interpretation of the non-significant pooled mean difference.

To assess the robustness of the three-level model, its fit was compared to that of equivalent two-level models; one with the variance at level 2 (within-study) and one with the variance at level 3 (between-study) fixed to zero. Likelihood ratio tests showed that the three-level model (AIC = 155.9, BIC = 159.4) demonstrated significantly better fit than the model constraining within-study variance (AIC = 213.8, BIC = 216.2, *χ*^2^_(1)_ = 59.90, *p* < 0.001), and also significantly better fit than the model constraining between-study variance (AIC = 166.2, BIC = 168.6, χ^2^_(1)_ = 12.33, *p* < 0.001). This suggests that significant variability existed in effect sizes within studies, as well as between studies. Therefore, the three-level model was retained because it accounts for statistical dependency in the data structure arising from the potentially overlapping cohorts (Figure 3). The implementation of the three-level model resulted in between-study heterogeneity of *I*^2^ = 95.7% and within-study heterogeneity of *I*^2^ = 4.3%.

### 3.4 Subgroup analysis

The analysis of potential moderators, including BMI, sex, diagnostic criteria (NAFLD, MAFLD, MASLD, or NASH), diagnostic methods (FLI, abdominal ultrasound, MRI, or liver biopsy), and Lp(a) measurement methods (immunoturbidimetry, nephelometry method, or not reported), did not significantly impact on circulating Lp(a) levels (Supplementary Tables 3.1–3.5).

### 3.5 Sensitivity analysis

The sensitivity analyses showed that omitting any single cluster did not yield a statistically significant summary effect (Supplementary Figure 3). The study by Sukahri et al. (28) emerged as a potential outlier, as demonstrated in Supplementary Figures 3 and 4.

The removal of four studies rated as ‘poor’ (25, 30, 32, 41) and two studies rated as ‘fair’ (33, 34) quality according to the NIH quality assessment resulted in increased MD from 1.40 to 1.96 mg/dL (95% CI: −3.73 to 7.67, *p* = 0.47), with the overall effect remaining statistically non-significant, while the between-study heterogeneity increased (*I*^2^ = 99.9%).

### 3.6 Publication bias

The Egger’s-type regression test indicated no statistically significant evidence of small-study effects (*β*_0_ = 0.66 [−0.64, 1.97], *p* = 0.31), suggesting no statistically significant funnel-plot asymmetry when accounting for dependence among effect sizes.

The power-enhanced sunset plot (Supplementary Figure 6) showed that 12 of the 21 studies included in the meta-analysis (27–29, 31, 34, 35, 38–42, 45) had statistical power greater than 15% to detect the meta-analytic summary effect. The median statistical power across all studies was very low 19.7%.

A p-curve analysis was conducted on 16 statistically significant results (*p* < 0.05), 13 of which were highly significant (*p* < 0.025). As illustrated in Supplementary Figure 7, the findings collectively suggest that selective reporting or data manipulation was unlikely. This is supported by significant right-skewness tests (*p* < 0.05), nonsignificant flatness tests (*p* > 0.05), and high estimated power (99%). Although the possibility of researcher bias cannot be entirely excluded, the results of the p-curve analysis provide evidence that the observed summary effect is unlikely to be attributable to selective reporting or p-hacking.

## 4 Discussion

To date, the significance of Lp(a) as a CVD-related biomarker is consistently growing, but there are inconsistent findings from individual clinical studies on circulating Lp(a) in patients with metabolic-related steatosis/steatohepatitis (MASLD or NAFLD or MAFLD) (25–28, 32–40, 68–70). As such, the present systematic review and meta-analysis was conducted to consolidate the available literature through a comprehensive evidence synthesis of relevant clinical data. Indeed, the current findings provide the most up-to-date and robust inferences regarding the biomarker potential of circulating Lp(a) in adults with metabolic-related steatosis/steatohepatitis. In contrast to two previous systematic reviews on this topic which did not include a meta-analysis (71, 72), the present systematic review includes a meta-analysis which showed that, based on the 21 identified eligible studies, circulating Lp(a) concentrations exhibit no significant differences in adults with MASLD or NAFLD or MAFLD compared to controls. To our knowledge, there is only one previous meta-analysis on this research question by Nikoli et al. (73), which covered studies up to 2024 and reported similar findings to our meta-analysis. However, the three-level meta-analysis structure which we applied to our data avoided errors that occur with conventional reductionist approaches to meta-analysis. This provided a more robust framework for analysing the differences of Lp(a) levels between the cases and controls (74). Of note, although the overall difference was not statistically significant, the applied model successfully partitioned variability, identifying substantial heterogeneity primarily between, rather than within individual studies (75). Furthermore, the present analyses showed that sex, BMI, diagnostic criteria for steatosis/steatohepatitis, steatosis/steatohepatitis diagnostic methods, and Lp(a) measurement methods were not significant moderators of the circulating Lp(a) levels. It is noteworthy that circulating Lp(a) concentrations are primarily governed by variation at the *LPA* locus, particularly the kringle-IV (KIV) type 2 repeat number that determines apolipoprotein(a) isoform size, which accounts for most inter-individual variability (76). Indeed, circulating Lp(a) concentrations remain relatively stable during adulthood, with the exception of certain diseases (e.g., certain endocrine diseases and chronic kidney disease), and are narrowly influenced by lifestyle factors (e.g., by diet or exercise) (77).

A sensitivity analysis was also conducted in the present study to assess the robustness of the overall effect size and to identify influential studies that may have contributed to heterogeneity. The exclusion of the study by Sukahri et al. (28), as a potential outlier, resulted in a reduction in heterogeneity but did not result in a statistically significant change in the summary effect. Indeed, the study by Sukahri et al. is a small study which reported that patients with NAFLD had higher levels of Lp(a) compared to controls (28). Although both the NAFLD (*n* = 90) and control (*n* = 20) groups of the study by Sukahri et al. (28) had obstructive sleep apnoea, which is a factor that may substantially contribute to inflammatory and metabolic dysregulation (78), the small sample size of that study raises concerns about the potential for random variation, which may undermine the representation of a genuine underlying effect (79).

### 4.1 Potential Lp(a) and MASLD associations

The precise role of Lp(a) in the pathophysiology of MASLD has yet to be fully elucidated, since, although many aspects of Lp(a) metabolism are well-studied, other aspects remain largely unclear (80). Lp(a) is an LDL-like particle primarily synthesized in the liver with a single apolipoprotein B100 covalently linked by a disulfide bond to a single apolipoprotein(a) (11–14). Evidence suggests that Lp(a) levels are reduced in the context of hepatocellular damage, particularly in conditions such as chronic active hepatitis C, primary biliary cholangitis, and hepatocellular carcinoma (81–83). Additionally, a study conducted by Konishi et al. in Japan demonstrated that circulating Lp(a) concentrations were inversely correlated with the degree of advanced hepatic fibrosis in patients with biopsy-proven NASH (68). Similarly, a study conducted by Meroni et al. in Italy revealed that patients with biopsy-proven NAFLD and low circulating Lp(a) concentrations exhibited increased risk of developing severe fibrosis and cirrhosis (70). Furthermore, in a cohort of 151 patients with biopsy-confirmed NAFLD/NASH, circulating Lp(a) concentrations were markedly lower in individuals with NASH compared to those with NAFLD who did not have NASH (84). In that study, circulating Lp(a) concentrations had an inverse association with key histopathological features, including hepatocyte ballooning, lobular inflammation, and fibrosis (84). These low circulating Lp(a) concentrations in advanced liver fibrosis and cirrhosis could be, at least in part, explained by a reduction in the hepatic synthesis of Lp(a) (28). However, these studies (68, 70, 84) were not eligible for inclusion in the present systematic review due to lacking a control group, which was considered an important inclusion criterion in order to exclude studies more prone to bias and confounding (85).

Moreover, although Lp(a) concentrations in adults are generally regarded as relatively stable and are not influenced by the fasting status (86), these may be influenced by factors such as storage temperature (87). Accordingly, there are data indicating that storage conditions, including temperature, storage duration, and the number of freezing–thawing cycles, can influence Lp(a) measurements and potentially lead to falsely low Lp(a) levels in epidemiological studies (88). For example, this issue has been observed in studies examining the association between Lp(a) and coronary artery disease, where lower circulating Lp(a) concentrations in patients with coronary artery disease compared to controls have historically delayed establishing a definitive relationship between elevated Lp(a) concentrations and the development and progression of coronary artery disease (88). Regarding Lp(a) measurement methods, the analyses performed for the present meta-analysis revealed no significant differences between the utilized nephelometric and immunoturbidimetric Lp(a) measurement methods. This finding can be attributed, at least in part, to the fact that both methods utilize the same polyclonal antibodies against apolipoprotein(a) (89). However, the Lp(a) measurement method was not clearly specified in five of the included studies (29, 30, 33, 40, 43), whilst detailed storage conditions of the analysed samples (e.g., storage duration and number of freezing–thawing cycles) were not reported by the included studies. In addition, the KIV repeat structure of apo(a) may result in a measurement bias known as apo(a) isoform-sensitive assays, meaning that serum concentrations of large isoforms with a high number of KIV repeats are typically associated with low Lp(a) levels, whereas small isoforms with fewer KIV repeats are associated with higher Lp(a) levels (90).

Furthermore, ethnicity appears to influence Lp(a) levels, with those of Asian and European background exhibiting lower levels compared to individuals of African descent (91). In the present systematic review, the majority of the included cohorts were from Asia, with limited studies/cohorts from Europe. This is in line with the global distribution of studies included in the previous relevant systematic reviews, such as the one by Kouvari et al., where the majority of included studies were also from Asia, with one study from Europe (72). Given this paucity of ethnicity-related data, the potential impact of ethnicity on circulating Lp(a) in the context of MASLD merits targeted research to elucidate any relevant underlying differences in patients of different ethnic backgrounds.

### 4.2 Circulating Lp(a) concentration and CVD within the context of steatotic liver disease

Among the studies where subclinical CVD was assessed using surrogate markers [e.g., carotid intima-media thickness (CIMT), silent myocardial ischaemia via electrocardiogram, and coronary artery calcium score via computed tomography coronary angiography], Sukahri et al. (28) reported that a markedly higher proportion of patients with NAFLD had CIMT >0.80 mm (64.4%) compared to controls (5%), with significantly higher circulating Lp(a) concentrations. Moreover, Wu et al. (31) reported that patients with NAFLD had higher subclinical ischaemic heart disease (IHD) (10.8%) compared to controls (5.4%), with significantly higher circulating Lp(a) concentrations. In contrast, Lee et al. (44) reported increased silent myocardial ischaemia in patients with NAFLD (12%) compared to controls (1%), despite paradoxically lower circulating Lp(a) concentrations in the NAFLD group. However, regarding clinical CVD, within the cohort of Sukahri et al. (28), the NAFLD group also demonstrated a higher prevalence of IHD (22.22%) relative to the non-NAFLD group (15%) with significantly higher circulating Lp(a) concentrations compared to controls. In addition, patients with NAFLD within the cohort of Wu et al. (31) had higher unstable angina, myocardial infarction, and resuscitated cardiac arrest (14.3%) compared to controls (6.5%), with significantly higher circulating Lp(a) concentrations. Furthermore, Lin et al. (29) reported a significantly higher prevalence of IHD among patients with NAFLD (21.6%) compared to controls (14.7%) along with elevated circulating Lp(a) concentrations. Notably, in a large prospective cohort of 56,168 patients with MASLD followed for a median of 5 years, 6,136 individuals developed incident major adverse cardiovascular events (MACE) (92). The study demonstrated that MASLD patients with advanced fibrosis and elevated Lp(a) levels had a significantly higher risk of MACE compared to those with lower Lp(a) levels (adjusted hazard ratio = 1.56, 95% CI: 1.27 to 1.91; *p* < 0.001) (92). These findings highlight the need for further research to elucidate the relationship between circulating Lp(a) and CVD risk in the context of steatotic liver disease related to metabolic dysfunction and to determine whether Lp(a) can be reliably used for risk stratification in this patient population.

### 4.3 Study strengths and comparison to previous systematic reviews/meta-analysis

The present systematic review and meta-analysis has notable strengths, including a meticulous study identification process from the most commonly used biomedical databases and a robust evidence synthesis and quality assessment. Accordingly, to our knowledge, the present work offers the most up-to-date meta-analysis on circulating Lp(a) concentrations in adults with MASLD or NAFLD or MAFLD compared to controls with the largest number of studies (*n* = 21) and largest sample size (137,494 cases and 281,261 controls) analysed so far. It is also noteworthy that all included studies had a control group; thus the circulating Lp(a) concentrations between cases and controls were validated within individual studies, and so the present meta-analysis avoided the potential bias/limitation which could be introduced by including studies without control groups (85). Furthermore, the application of a three-level random-effects model and sensitivity analysis enhances the robustness and reliability of the present findings. Finally, the comprehensive evaluation of the potential publication bias further strengthens the validity of these results. Of note, while publication bias may have impacted some of the outcomes in the current meta-analysis, p-curve analysis indicates that the observed overall effect is true and unlikely to be a consequence of selective reporting or data manipulation. However, it is important to recognize that publication bias may not arise solely from effect sizes but may also be influenced by *p*-values (54, 55, 75). Based on these strengths, the present work not only updates the previous systematic reviews/meta-analysis on this topic, but also offers a robust analysis based on a larger number of studies and participants, as summarized in Table 3.

**Table 3:** Overview of the present systematic review and meta-analysis compared to previous systematic reviews with or without a meta-analysis on the potential relationship between circulating Lp(a) concentrations and steatotic liver disease related to metabolic dysfunction.

Comparison	Current study (2025)	Nikoli et al. (73)	Masson et al. (71)	Kouvari and Mantzoros (72)
Study design	Systematic review and meta-analysis	Systematic review and meta-analysis	Systematic review without meta-analysis	Systematic review without meta-analysis
Inclusion criteria	Observational studies which assessed circulating Lp(a) concentrations in adults (≥18 years of age) diagnosed with MASLD or MAFLD or NAFLD or NASH in comparison to control groups without such hepatic pathology.	Observational studies with quantitative measurement of circulating Lp(a) concentrations for patients with NAFLD (or with appropriately defined patients with MAFLD) and controls without NAFLD.	Observational studies that have evaluated Lp(a) levels in a group of adult patients with NAFLD (patients classified as NASH or MAFLD were also considered) compared to a control group.	Research studies on the association between Lp(a) and overall NAFLD or specific elements of NAFLD (e.g., liver steatosis, liver fibrosis or liver enzymes) were eligible. Eligible studies included original research articles retrieved from prospective studies (cohort studies or case-cohort studies) or retrospective or cross-sectional studies with ≥100 participants.
Number of included studies	21	18	10	9
Number of included cases/controls	137,494 cases; 281,261 controls	20,220 cases; 54,471 controls	9,266 cases; 30,779 controls	16,564 cases; 33,295 controls
Key findings	Circulating Lp(a) concentrations were not significantly different between adults with MASLD or MAFLD or NAFLD/NASH and controls without such a hepatic pathology.	Circulating Lp(a) was similar between NAFLD patients and non-NAFLD controls. Patients with NAFLD had lower circulating Lp(a) compared with controls, when Lp(a) was measured with nephelometry.	Although a potential link between Lp(a) levels and hepatic steatosis has been suggested, existing evidence is inconsistent.	Most of the included studies revealed a significant inverse association between Lp(a) and liver fibrosis.

Lp(a), Lipoprotein(a); MAFLD, metabolic dysfunction-associated fatty liver disease; MASLD, metabolic dysfunction-associated steatotic liver disease; NAFLD, non-alcoholic fatty liver disease; NASH: non-alcoholic steatohepatitis.

### 4.4 Study limitations

Despite the aforementioned strengths, certain limitations must also be acknowledged in the context of the present analyses. Firstly, although we analysed the largest number of eligible studies so far, the number of relevant eligible studies remains relatively low, which potentially limits the overall strength of the present findings. For example, most eligible studies were from Asia, with an even more limited number from Europe, which restricts the generalisability of the findings to other regions and highlights the need for further population-based research in this field across diverse geographic areas. Furthermore, all eligible studies were observational with a cross-sectional design, which precludes inferences on causality (93). Observational studies are also considered to produce lower quality evidence, including a greater potential bias (94). In addition, meta-regression analyses using aggregate study-level variables, such as the mean BMI and the proportion of male participants, cannot be interpreted as evidence of individual-level associations. These analyses are study-level exploratory meta-regressions and the coefficients reflect between-study differences which should not be interpreted as higher BMI or being male at the individual participant level is directly associated with the observed effect size. Of note, although each eligible study included a control group, caution is warranted, as undetermined phenotypes may be concealed within the demographic characteristics (85). In addition, relevant publications in other languages may have been missed by including only English-language papers, and it is not possible to definitely determine whether excluding languages other than English might have introduced a systematic language bias in the present systematic review (95). The lack of standardization in measuring circulating Lp(a) across studies may have also introduced variability and inaccuracy in reported values (96). Differences in reported Lp(a) units (nmol/L versus mg/dL) required the application of a conversion factor (0.465) to harmonize data for a limited subset of studies (five of 21 studies). While this approach enabled the inclusion of all relevant studies in the pooled analysis, it does not account for inter-individual variability in apolipoprotein(a) isoform size, which can affect the molecular weight of Lp(a) particles (96). Consequently, these converted values should be regarded as approximations, and this limitation should be considered when interpreting the findings. Furthermore, the Lp(a) measurement method was not reported in a five of the included eligible studies (Table 2) and, despite the performed subgroup analysis by measurement method, it is unclear how this can impact on the reliability of the pooled estimates. Moreover, the inclusion of multiple effect sizes from the same cohort (e.g., UK Biobank) may have influenced the pooled estimates. Although a multilevel model was applied to account for this, some residual over-representation cannot be excluded. It should be also noted that grouping together distinct histological stages of NAFLD, MAFLD or MAFLD, from simple steatosis to advanced steatohepatitis, into broad categories within some included cohorts might obscure subtle but important variations in circulating Lp(a) concentrations that may occur during disease progression.

Finally, substantial heterogeneity was observed among the eligible studies, possibly driven by multifactorial interactions and unmeasured variables. Although unavoidable, this further highlights the critical need for additional large studies in this field which should cover a wider geographical area using standardized methodological procedures. In this context, future studies in this field should apply consistent Lp(a) measurement protocols and reporting, which would ensure accurate and comparable results across studies. Future research should evaluate the reliability and clinical interpretability of circulating Lp(a) in patients with steatotic liver disease, particularly in the context of CVD. These investigations will strengthen the clinical interpretability, generalisability, and translational relevance of Lp(a) as a biomarker in this patient population.

## 5 Conclusion

To our knowledge, this systematic review and meta-analysis study provides the most up-to-date and comprehensive evidence synthesis on the potential correlation between circulating Lp(a) and steatotic liver disease related to metabolic dysfunction. Based on the largest number of studies/participants analysed so far, the present findings suggest that there is no significant difference in circulating Lp(a) concentrations between adults with NAFLD or MAFLD or MASLD and controls without such a liver disease. This suggests that the potential for circulating Lp(a) as a diagnostic/prognostic biomarker for MASLD is limited. However, given its established role in CVD, Lp(a) could still be utilized to evaluate CVD risk in the context of steatotic liver disease. Thus, future prospective studies should also focus on exploring the clinical utility of circulating Lp(a) as a biomarker for long-term CVD outcomes in MASLD. Advancing knowledge in this area can contribute to refine the cardio-metabolic risk stratification, promote more tailored monitoring approaches and enhance the clinical management of this increasingly prevalent patient population.

## Data Availability

The original contributions presented in the study are included in the article/Supplementary material, further inquiries can be directed to the corresponding authors.
