# Supplementary material for: Circulating lipoprotein(a) levels and steatotic liver disease related to metabolic dysfunction in adults: an updated systematic review and meta-analysis
Source: Front Nutr. 2026 May 8;13:1778393. doi: 10.3389/fnut.2026.1778393 (PMC13193877; doi:10.3389/fnut.2026.1778393)
Supplement: Supplementary file 1 [file Supplementary_file_1.docx]

**Supplementary Materials**

**Supplementary Table 1.** The Preferred Reporting Items for Systematic Reviews and Meta-Analyses (PRISMA) checklist for the present systematic review.

| **Section and Topic** | **Item #** | **Checklist item** | **Location where item is reported** |
| --- | --- | --- | --- |
| **TITLE** | | |  |
| Title | 1 | Identify the report as a systematic review. | Title page |
| **ABSTRACT** | | |  |
| Abstract | 2 | See the PRISMA 2020 for Abstracts checklist. | Abstract |
| **INTRODUCTION** | | |  |
| Rationale | 3 | Describe the rationale for the review in the context of existing knowledge. | Section 1 |
| Objectives | 4 | Provide an explicit statement of the objective(s) or question(s) the review addresses. | Section 1 |
| **METHODS** | | |  |
| Eligibility criteria | 5 | Specify the inclusion and exclusion criteria for the review and how studies were grouped for the syntheses. | Section 2.3 |
| Information sources | 6 | Specify all databases, registers, websites, organisations, reference lists and other sources searched or consulted to identify studies. Specify the date when each source was last searched or consulted. | Section 2.2 |
| Search strategy | 7 | Present the full search strategies for all databases, registers and websites, including any filters and limits used. | Supplementary materials |
| Selection process | 8 | Specify the methods used to decide whether a study met the inclusion criteria of the review, including how many reviewers screened each record and each report retrieved, whether they worked independently, and if applicable, details of automation tools used in the process. | Section 2.4 |
| Data collection process | 9 | Specify the methods used to collect data from reports, including how many reviewers collected data from each report, whether they worked independently, any processes for obtaining or confirming data from study investigators, and if applicable, details of automation tools used in the process. | Section 2.4 |
| Data items | 10a | List and define all outcomes for which data were sought. Specify whether all results that were compatible with each outcome domain in each study were sought (e.g. for all measures, time points, analyses), and if not, the methods used to decide which results to collect. | Section 2.4 |
|  | 10b | List and define all other variables for which data were sought (e.g. participant and intervention characteristics, funding sources). Describe any assumptions made about any missing or unclear information. | Section 2.4 |
| Study risk of bias assessment | 11 | Specify the methods used to assess risk of bias in the included studies, including details of the tool(s) used, how many reviewers assessed each study and whether they worked independently, and if applicable, details of automation tools used in the process. | Section 2.5 |
| Effect measures | 12 | Specify for each outcome the effect measure(s) (e.g. risk ratio, mean difference) used in the synthesis or presentation of results. | Section 2.6 |
| Synthesis methods | 13a | Describe the processes used to decide which studies were eligible for each synthesis (e.g. tabulating the study intervention characteristics and comparing against the planned groups for each synthesis (item #5)). | Section 2.6 |
|  | 13b | Describe any methods required to prepare the data for presentation or synthesis, such as handling of missing summary statistics, or data conversions. | Section 2.6 |
|  | 13c | Describe any methods used to tabulate or visually display results of individual studies and syntheses. | Section 2.6 |
|  | 13d | Describe any methods used to synthesize results and provide a rationale for the choice(s). If meta-analysis was performed, describe the model(s), method(s) to identify the presence and extent of statistical heterogeneity, and software package(s) used. | Section 2.6 |
|  | 13e | Describe any methods used to explore possible causes of heterogeneity among study results (e.g. subgroup analysis, meta-regression). | Section 2.6 |
|  | 13f | Describe any sensitivity analyses conducted to assess robustness of the synthesized results. | Section 2.6 |
| Reporting bias assessment | 14 | Describe any methods used to assess risk of bias due to missing results in a synthesis (arising from reporting biases). | Section 2.6 |
| Certainty assessment | 15 | Describe any methods used to assess certainty (or confidence) in the body of evidence for an outcome. | Section 2.6 |
| **RESULTS** | | |  |
| Study selection | 16a | Describe the results of the search and selection process, from the number of records identified in the search to the number of studies included in the review, ideally using a flow diagram. | Section 3.1 |
|  | 16b | Cite studies that might appear to meet the inclusion criteria, but which were excluded, and explain why they were excluded. | N/A |
| Study characteristics | 17 | Cite each included study and present its characteristics. | Section 3.1  Table 2 |
| Risk of bias in studies | 18 | Present assessments of risk of bias for each included study. | Section 3.2 |
| Results of individual studies | 19 | For all outcomes, present, for each study: (a) summary statistics for each group (where appropriate) and (b) an effect estimate and its precision (e.g. confidence/credible interval), ideally using structured tables or plots. | Section 3.3 |
| Results of syntheses | 20a | For each synthesis, briefly summarise the characteristics and risk of bias among contributing studies. | Section 3.3 |
|  | 20b | Present results of all statistical syntheses conducted. If meta-analysis was done, present for each the summary estimate and its precision (e.g. confidence/credible interval) and measures of statistical heterogeneity. If comparing groups, describe the direction of the effect. | Section 3.4-3.6 |
|  | 20c | Present results of all investigations of possible causes of heterogeneity among study results. | Section 3.4-3.6 |
|  | 20d | Present results of all sensitivity analyses conducted to assess the robustness of the synthesized results. | Section 3.4-3.6 |
| Reporting biases | 21 | Present assessments of risk of bias due to missing results (arising from reporting biases) for each synthesis assessed. | N/A |
| Certainty of evidence | 22 | Present assessments of certainty (or confidence) in the body of evidence for each outcome assessed. | N/A |
| **DISCUSSION** | | |  |
| Discussion | 23a | Provide a general interpretation of the results in the context of other evidence. | Section 4 |
|  | 23b | Discuss any limitations of the evidence included in the review. | Section 4 |
|  | 23c | Discuss any limitations of the review processes used. | Section 4 |
|  | 23d | Discuss implications of the results for practice, policy, and future research. | Section 4 |
| **OTHER INFORMATION** | | |  |
| Registration and protocol | 24a | Provide registration information for the review, including register name and registration number, or state that the review was not registered. | Section 2.1 |
|  | 24b | Indicate where the review protocol can be accessed, or state that a protocol was not prepared. | Section 2.1 |
|  | 24c | Describe and explain any amendments to information provided at registration or in the protocol. | N/A |
| Support | 25 | Describe sources of financial or non-financial support for the review, and the role of the funders or sponsors in the review. | Funding Section |
| Competing interests | 26 | Declare any competing interests of review authors. | Conflict of Interests section |
| Availability of data, code and other materials | 27 | Report which of the following are publicly available and where they can be found: template data collection forms; data extracted from included studies; data used for all analyses; analytic code; any other materials used in the review. | Data availability statement section |

*From:*  Page MJ, McKenzie JE, Bossuyt PM, Boutron I, Hoffmann TC, Mulrow CD, et al. The PRISMA 2020 statement: an updated guideline for reporting systematic reviews. BMJ 2021;372:n71. doi: 10.1136/bmj.n71.

**Supplementary Table 2.** Search strategy implemented across the Embase, CINAHL, and Scopus databases.

**Supplementary Table 2.1.** Search strategy used in Embase.

| 1 | ("Lipoprotein a" or "lipoprotein(a)" or "lipoprotein (a)" or "apoprotein a" or "apolipoprotein(a)" or "apolipoprotein (a)" or "apolipoprotein a" or "apolipoprotein(a)" or "apolipoprotein (a)" or "Lp a" or "Lp(a)" or "Lp (a)" or "apo a" or "apo(a)" or "apo (a)" or "APOA5").mp. |
| --- | --- |
| 2 | ("metabolic associated fatty liver disease".tw. or MAFLD.mp. or "metabolic dysfunction associated fatty liver disease".tw. or exp non alcoholic fatty liver disease/ or NAFLD.tw. or "non-alcoholic fatty liver disease".tw. or "non-alcoholic steatohepatitis".tw. or NASH.tw. or "metabolic dysfunction-associated steatotic liver disease".tw. or "fatty liver".ti. or MASLD.tw.) |
| 3 | 1 and 2 |

**Supplementary Table 2.2.** Search strategy used in CINAHL.

| S1 | ("Lipoprotein a" or "lipoprotein(a)" or "lipoprotein (a)" or "apoprotein a" or "apolipoprotein(a)" or "apolipoprotein (a)" or "apolipoprotein a" or "apolipoprotein(a)" or "apolipoprotein (a)" or "Lp a" or "Lp(a)" or "Lp (a)" or "apo a" or "apo(a)" or "apo (a)" or "APOA5").mp. |
| --- | --- |
| S2 | "metabolic associated fatty liver disease".tw. or MAFLD.mp. or "metabolic dysfunction associated fatty liver disease".tw. or exp non alcoholic fatty liver disease/ or NAFLD.tw. or "non-alcoholic fatty liver disease".tw. or "non-alcoholic steatohepatitis".tw. or NASH.tw. or "metabolic dysfunction-associated steatotic liver disease".tw. or "fatty liver".ti. or MASLD.tw. |
| S3 | S1 and S2 |

**Supplementary Table 2.3.** Search strategy used in Scopus.

(TITLE-ABS-KEY("Lipoprotein a" or "lipoprotein(a)" or "lipoprotein (a)" or "apoprotein a" or "apolipoprotein(a)" or "apolipoprotein (a)" or "apolipoprotein a" or "apolipoprotein(a)" or "apolipoprotein (a)" or "Lp a" or "Lp(a)" or "Lp (a)" or "apo a" or "apo(a)" or "apo (a)" or "APOA5"))

AND

(TITLE-ABS-KEY("metabolic associated fatty liver disease" or MAFLD or "metabolic dysfunction associated fatty liver disease" or NAFLD or "non-alcoholic fatty liver disease" or "non-alcoholic steatohepatitis" or NASH or "metabolic dysfunction-associated steatotic liver disease" or "fatty liver" or MASLD))

**Supplementary Table 3.**  Three-level meta-regression model estimating the moderating effects on circulating Lipoprotein(a) [Lp(a)] levels of:

**Supplementary Table 3.1.** Body mass index (BMI):

| Moderator | β | SE | 95% CI | P |
| --- | --- | --- | --- | --- |
| Intercept | 0.48 | 2.75 | (-5.22, 6.17) | .86 |
| BMI | 0.36 | 0.51 | (-0.69, 1.42) | .48 |
| Abbreviations: 95% CI: 95% confidence interval; β: estimate; BMI: body mass index; P: p-value; SE: standard error. | | | | |

**Supplementary Table 3.2.** Participants’ Sex:

| Moderator | β | SE | 95% CI | P |
| --- | --- | --- | --- | --- |
| Intercept | 1.95 | 2.07 | (-2.33, 6.23) | .35 |
| Female | 0.07 | 0.04 | (-0.01, 0.14) | .07 |
| Abbreviations: 95% CI: 95% confidence interval; β: estimate; P: p-value; SE: standard error. | | | | |

| Moderator | β | SE | 95% CI | P |
| --- | --- | --- | --- | --- |
| Intercept | 1.02 | 2.38 | (-3.92, 5.97) | .67 |
| MASLD | 1.08 | 2.81 | (-4.77, 6.93) | .71 |
| MAFLD | -1.44 | 1.93 | (-5.46, 2.57) | .46 |
| NASH | 4.67 | 5.32 | (-6.39, 15.74) | .39 |
| Abbreviations: 95% CI: 95% confidence interval; β: estimate; MASLD: metabolic dysfunction-associated steatotic liver disease; MAFLD: metabolic dysfunction-associated fatty liver disease; NASH: non-alcoholic steatohepatitis; P: p-value; SE: standard error.  The intercept represents the NAFLD group which was used as the reference group. | | | | |

**Supplementary Table 3.3.** Clinical diagnosis of steatosis/steatohepatitis (i.e., non-alcoholic fatty liver disease [NAFLD], or non-alcoholic steatohepatitis [NASH], or metabolic-associated fatty liver disease [MAFLD], or metabolic dysfunction-associated fatty liver disease [MASLD]):

| Moderator | β | SE | 95% CI | P |
| --- | --- | --- | --- | --- |
| Intercept | 1.38 | 2.51 | (-3.85, 6.62) | .59 |
| Diagnostic method  Liver biopsy | 4.61 | 5.47 | (-6.81, 16.03) | .41 |
| Diagnostic method  (MRI) | -2.53 | 2.23 | (-7.18, 2.12) | .27 |
| Diagnostic method  (FLI) | -1.80 | 3.06 | (-8.19, 4.59) | .56 |
| Abbreviations: 95% CI: 95% confidence interval; β: estimate; MRI: Magnetic Resonance Image; FLI: Fatty Liver Index; P: p-value; SE: standard error.  The intercept represents the ultrasound based diagnosis which was used as the reference group.  [Note: the diagnostic method was insufficiently described for the patients with NAFLD in the study by El-Gazar *et al.* (38) and was not included in this sub-group analysis] | | | | |

**Supplementary Table 3.4.** Diagnostic method for steatosis/steatohepatitis (i.e., FLI, abdominal ultrasound, MRI, or liver biopsy):

**Supplementary Table 3.5.** Lipoprotein(a) [Lp(a)] measurement method (i.e., immunoturbidimetry, nephelometry method or not reported):

| Moderator | β | SE | 95% CI | P |
| --- | --- | --- | --- | --- |
| Intercept | 1.15 | 2.30 | (-3.61, 5.92) | .62 |
| Lp(a) measurement method (Nephelometric method) | - 0.41 | 2.09 | (- 4.74, 3.92) | .85 |
| Lp(a) measurement method  (method not reported) | 1.67 | 1.81 | (-2.08, 5.42) | .37 |
| Abbreviations: 95% CI: 95% confidence interval; β: estimate; P: p-value; SE: standard error.  The intercept represents the ultrasound based diagnosis which was used as the reference group.  The intercept represents the immunoturbidimetry measurement method which was used as the reference group. | | | | |


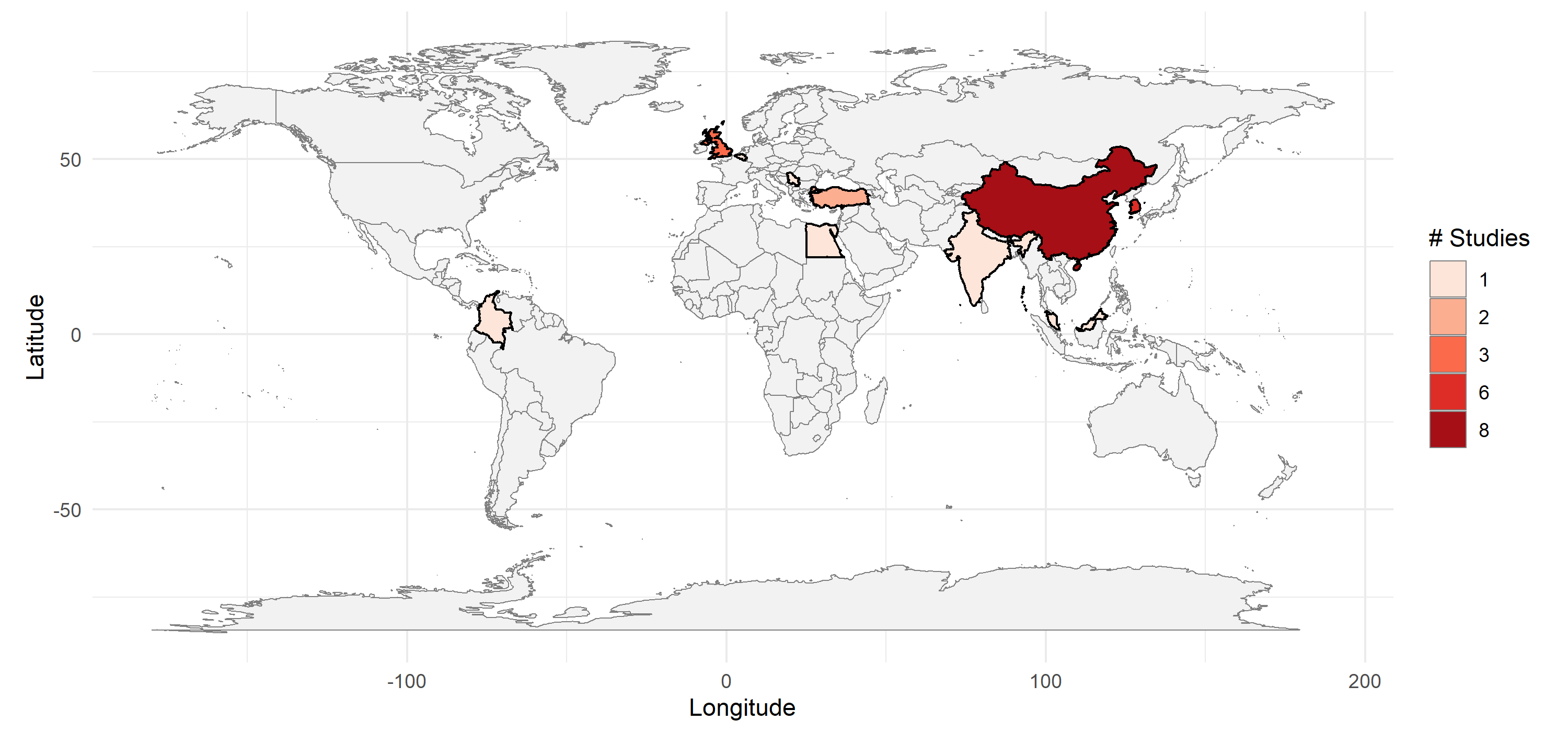


**Supplementary Figure 1**. Map of the world representing the countries from which cohorts originated in the included studies of this systematic review.

**
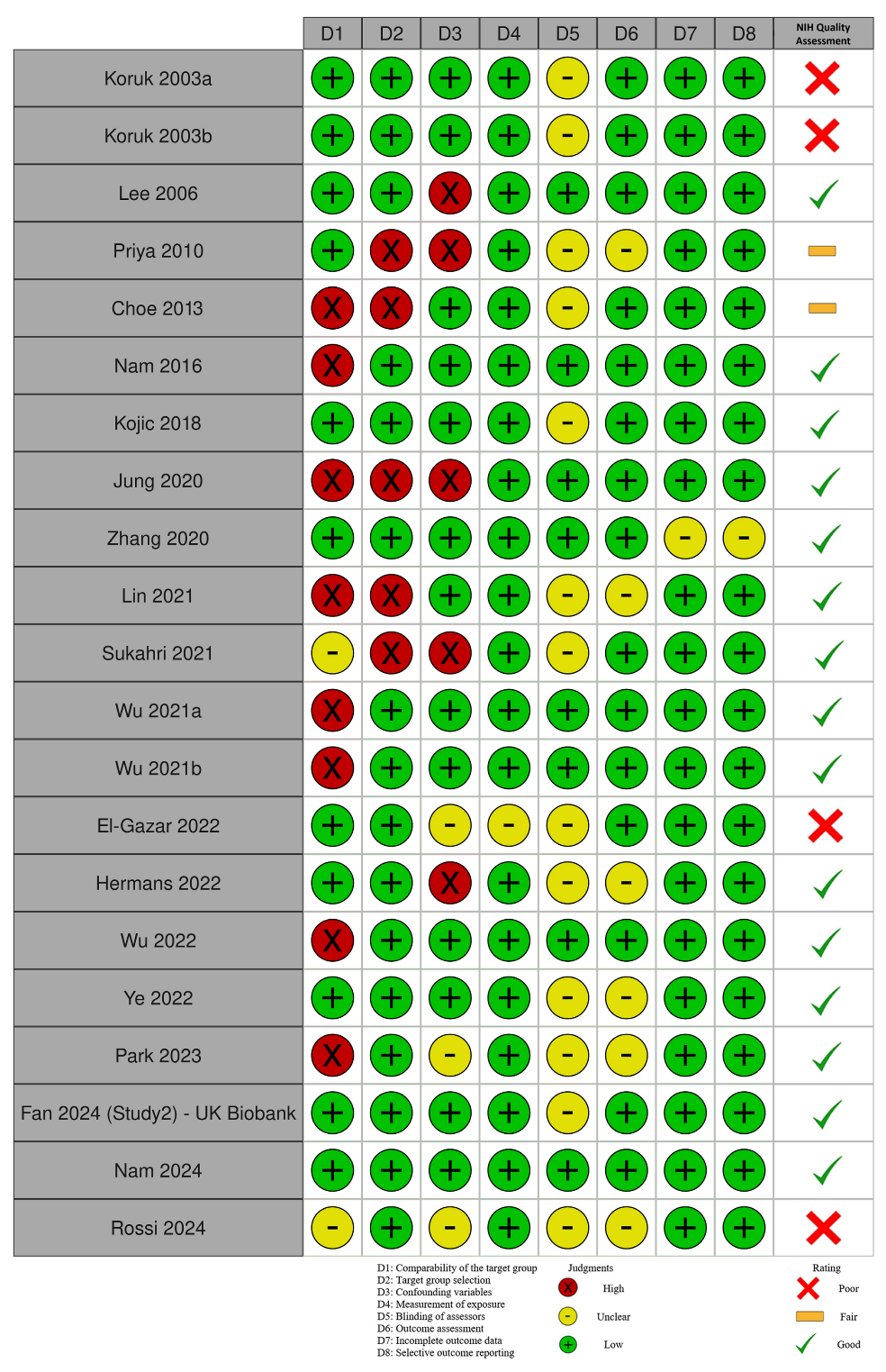
**

**Supplementary Figure 2**. Risk of Bias (RoB) assessment, using the Revised RoB Assessment Tool for Nonrandomized Studies (RoBANS 2), which includes eight domains (D1 to D8; i.e., comparability of the target group; target group selection; confounders; measurement of exposure; blinding of assessors; outcome assessment; incomplete outcome data; and selective outcome reporting) and quality assessment presented as good, fair or poor using the National Institute of Health (NIH) Study Quality Assessment Tool, for each of the 21 included studies in this systematic review. [Note: *Koruk 2023a* (Ref. 29) and *Koruk 2023b* (Ref. 22), and *Wu 2021a* (Ref. 35) and *Wu 2021b* (Ref. 28) correspond to the publications cited in this review].


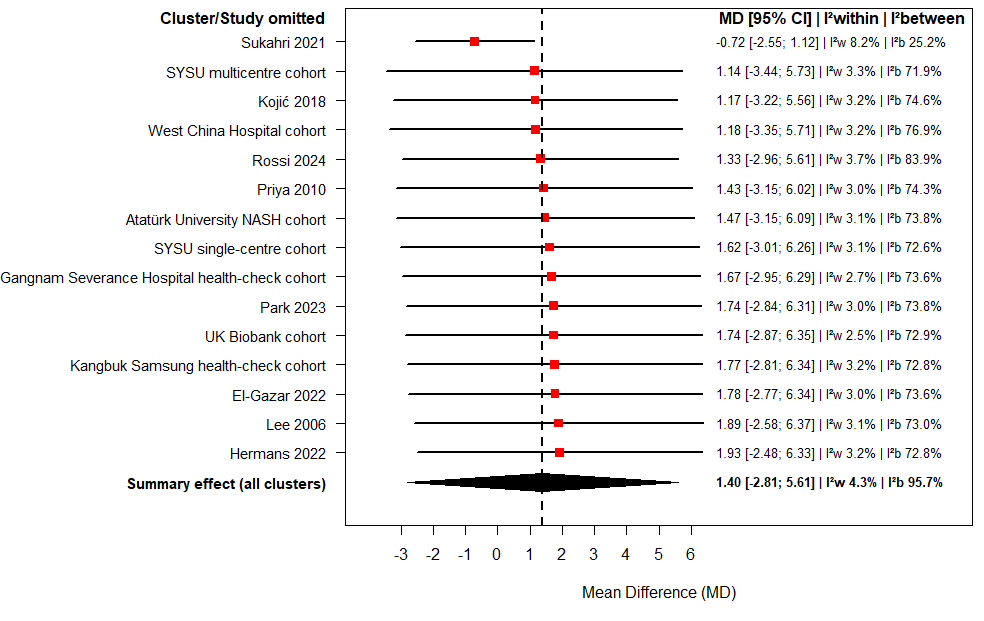


**Supplementary Figure 3.** Leave-one-out analysis at the cluster level with revised pooled estimates and heterogeneity within and between clusters. The applied clusters include: (i) the Sun Yat-sen University (SYSU), Guangdong, China multicenter cohort/cluster [cohort from Wu *et al.* 2021 (28), cohort from Lin *et al.* 2021 (26), and the Phase-2 Chinese cohort (validation cohort) from Ye *et al.* 2022 (24)]; (ii) the SYSU single-centre cohort/cluster [cohort from Wu *et al.* 2021 (35), the Phase-1 Chinese cohort from Ye *et al.* 2022 (24), and cohort from Wu *et al.* 2022 (36)]; (iii) the United Kingdom (UK) Biobank MAFLD cohort/cluster [the two UK Biobank cohorts/groups from Ye *et al.* 2022 (24), and cohort from Fan *et al.* 2024 (39)]; (iv) the Gangnam Severance Hospital health-check cohort/cluster [cohorts from Nam *et al.* 2016 (32), and Nam *et al.* 2024 (42)]; (v) the Kangbuk Samsung Hospital health-check cohort/cluster [cohorts from Choe *et al.* 2013 (31), and from Jung *et al.* 2020 (34)]; (vi) the Atatürk University NASH cohort/cluster [cohorts from the two studies by Koruk *et al.* (29) and (22)]; and (vii) the West China Hospital cohort/cluster including the NAFLD and NASH groups from the Zhang *et al.* 2020 (23) study which represented a single cohort that was categorized to control, NAFLD, and NASH groups.


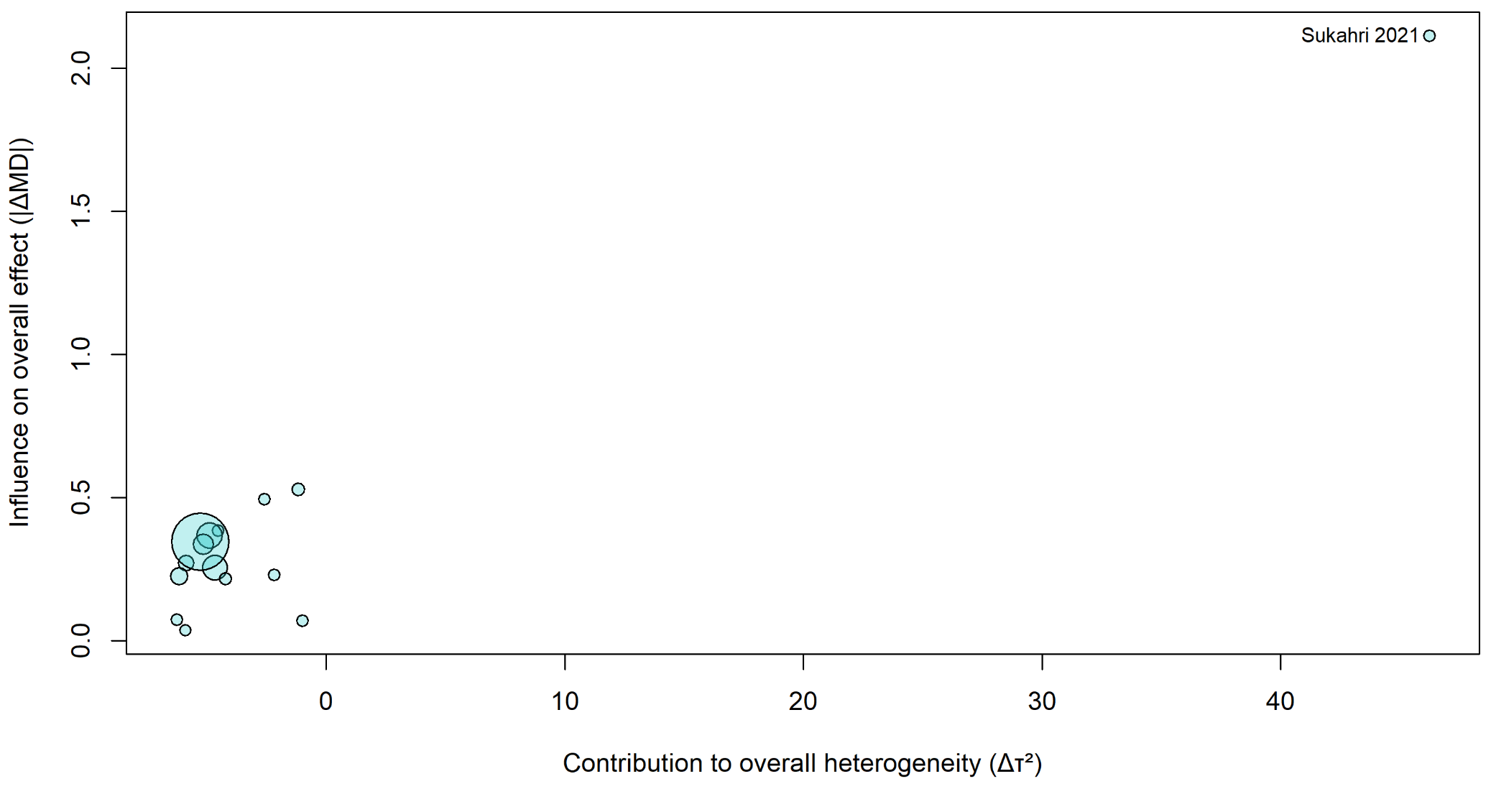


**Supplementary Figure 4**. Baujat plot of clusters. Clusters (or individual studies that formed their own cluster of k = 1) located away from the others were considered influential. Sukahri *et al.*, 2021 (25) was identified as a potential outlier. The applied clusters include: (i) the Sun Yat-sen University (SYSU), Guangdong, China multicenter cohort/cluster [cohort from Wu *et al.* 2021 (28), cohort from Lin *et al.* 2021 (26), and the Phase-2 Chinese cohort (validation cohort) from Ye *et al.* 2022 (24)]; (ii) the SYSU single-centre cohort/cluster [cohort from Wu *et al.* 2021 (35), the Phase-1 Chinese cohort from Ye *et al.* 2022 (24), and cohort from Wu *et al.* 2022 (36)]; (iii) the United Kingdom (UK) Biobank MAFLD cohort/cluster [the two UK Biobank cohorts/groups from Ye *et al.* 2022 (24), and cohort from Fan *et al.* 2024 (39)]; (iv) the Gangnam Severance Hospital health-check cohort/cluster [cohorts from Nam *et al.* 2016 (32), and Nam *et al.* 2024 (42)]; (v) the Kangbuk Samsung Hospital health-check cohort/cluster [cohorts from Choe *et al.* 2013 (31), and from Jung *et al.* 2020 (34)]; (vi) the Atatürk University NASH cohort/cluster [cohorts from the two studies by Koruk *et al.* (29) and (22)]; and (vii) the West China Hospital cohort/cluster including the NAFLD and NASH groups from the Zhang *et al.* 2020 (23) study which represented a single cohort that was categorized to control, NAFLD, and NASH groups.


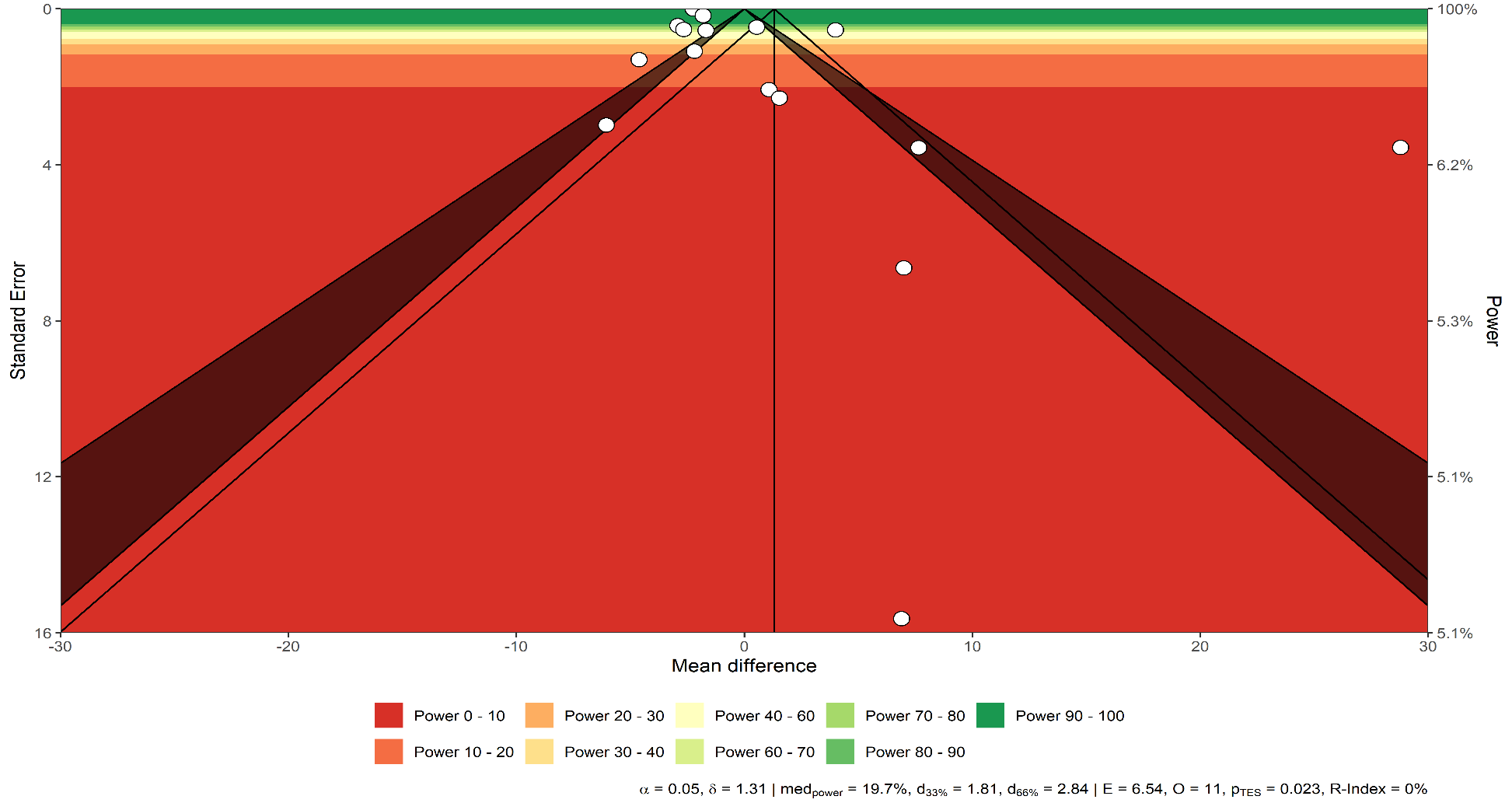


**Supplementary Figure 6.** A contour-enhanced funnel plot. The x axis shows the pooled effect size (Hedge’s g), the left y axis shows the standard error of the effect, and colours boundaries depict the statistical power of included studies. Power is computed at the study level assuming the meta-analytic summary effect as the true effect. Shaded contours represent 95% and 99% confidence intervals for an effect equal to zero. Alpha = 0.05, true effect size is 1.31, and median power of all the tests = 19.7%. The true effect size needed for achieving 33% and 66% of median power is 1.81 and 2.84, respectively. The R-index was 0%, suggesting that these studies unlikely of being replicated.


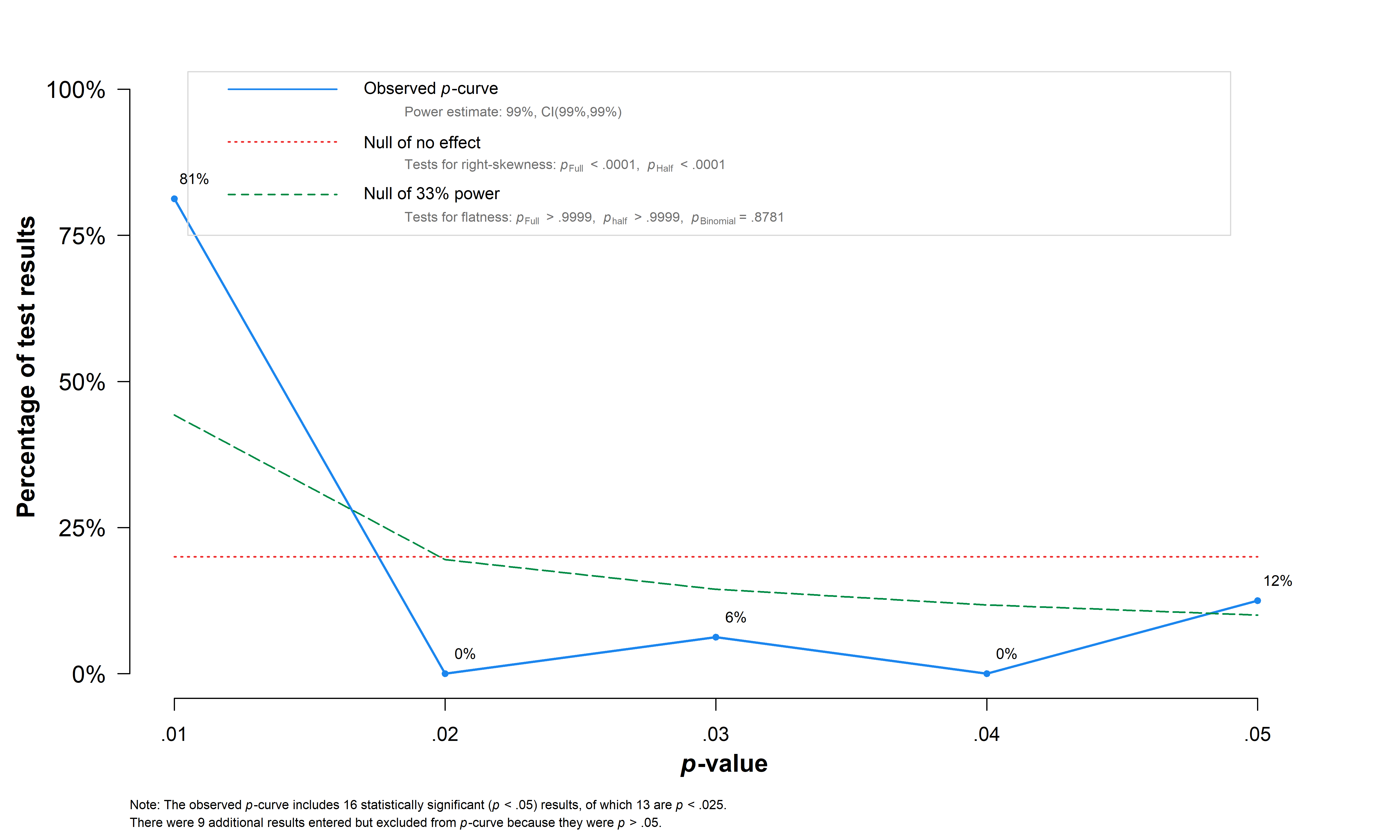


**Supplementary Figure 7**. The p-curve shows right skew, indicating that data manipulation and selective reporting bias were not present in the studies included in this systematic review.
